# Robust Ruthenium Catalysts Supported on Mesoporous Cyclodextrin-Templated TiO_2_-SiO_2_ Mixed Oxides for the Hydrogenation of Levulinic Acid to γ-Valerolactone

**DOI:** 10.3390/ijms22041721

**Published:** 2021-02-09

**Authors:** Cédric Decarpigny, Sébastien Noël, Ahmed Addad, Anne Ponchel, Eric Monflier, Rudina Bleta

**Affiliations:** 1 University Artois, CNRS, Centrale Lille, ENSCL, Univ. Lille, UMR 8181-UCCS-Unité de Catalyse et Chimie du Solide, F-62300 Lens, France; cedric.decarpigny@univ-artois.fr (C.D.); sebastien.noel@univ-artois.fr (S.N.); anne.ponchel@univ-artois.fr (A.P.); eric.monflier@univ-artois.fr (E.M.); 2 University Lille, CNRS, INRA, ENSCL, UMR 8207-UMET-Unité Matériaux et Transformations, F-59000 Lille, France; ahmed.addad@univ-lille.fr

**Keywords:** levulinic acid, γ-valerolactone, randomly methylated β-cyclodextrin, sol-gel, mixed oxides, Ru catalyst, aqueous phase hydrogenation

## Abstract

In this paper, we present a versatile template-directed colloidal self-assembly method for the fabrication in aqueous phase of composition-tuned mesoporous RuO_2_@TiO_2_-SiO_2_ catalysts. Randomly methylated β-cyclodextrin/Pluronic F127 supramolecular assemblies were used as soft templates, TiO_2_ colloids as building blocks, and tetraethyl orthosilicate as a silica source. Catalysts were characterized at different stages of their synthesis using dynamic light scattering, N_2_-adsorption analysis, powder X-ray diffraction, temperature programmed reduction, high-resolution transmission electron microscopy, high-angle annular bright-field and dark-field scanning transmission electron microscopy, together with EDS elemental mapping. Results revealed that both the supramolecular template and the silica loading had a strong impact on the pore characteristics and crystalline structure of the mixed oxides, as well as on the morphology of the RuO_2_ nanocrystals. Their catalytic performance was then evaluated in the aqueous phase hydrogenation of levulinic acid (LA) to γ-valerolactone (GVL) under mild conditions (50 °C, 50 bar H_2_). Results showed that the cyclodextrin-derived catalyst displayed almost quantitative LA conversion and 99% GVL yield in less than one hour. Moreover, this catalyst could be reused at least five times without loss of activity. This work offers an effective approach to the utilization of cyclodextrins for engineering the surface morphology of Ru nanocrystals and pore characteristics of TiO_2_-based materials for catalytic applications in hydrogenation reactions.

## 1. Introduction

With the growing concerns of accelerated global warming that the world faces today, the production of alternative fuels and renewable chemicals from biomass is an utmost challenge for the global scientific community. In this regard, the search for efficient catalytic routes and sustainable chemical processes to convert biomass into high value chemicals in a cost-effective and environmentally friendly manner is an essential step in the development of biorefinery technologies [1,2,3].

Among biomass-derived compounds, levulinic acid (LA) has been identified as an important platform chemical that can be obtained from the dehydration of lignocellulose-derived C6 sugars in the presence of mineral acids or ionic liquids. The hydrogenation and subsequent dehydration of LA produces γ-valerolactone (GVL) as its major product, which is considered as a sustainable fuel additive for the replacement of ethanol in ethanol/gasoline blends, in addition to its potential applications in pharmaceutical, perfume, and food industries, as well as its wide uses as a green solvent for biomass conversion [4].

The catalytic reduction of LA with hydrogen was first reported in 1930 by Schuette and Thomas [5], who showed that an 87% yield in GVL could be achieved in organic solvent with a PtO_2_ catalyst after 44 h at room temperature and 3 bar H_2_. Since then, many metal catalysts have been reported in the literature and among them, Ru has proven to be the most suitable for the hydrogenation of LA to GVL in aqueous phase [6,7,8,9,10,11], whereas Pd and Pt were found to be more active in the hydrogenation of ketones under gas phase conditions [12]. Michel et al. [13] correlated the better activity of Ru in aqueous phase to its higher degree of oxyphilicity with respect to Pd and Pt. Indeed, by combining DFT calculations and experimental data, it was shown that the interaction of water molecules with the carbonyl groups of LA adsorbed on the Ru surface could considerably lower the energy barrier of the reaction pathway, facilitating the hydrogenation of carbonyl groups [13,14,15]. Moreover, the increase in the amount of hydrogen atoms resulting from water dissociation on the Ru (0001) surface was identified as an additional factor responsible for the increased reaction rates [16]. 

The catalytic performance of Ru-based catalysts can be further improved by depositing the metal particles on a solid support. In this regard, mesoporous materials have been widely used as versatile supports owing to their high surface areas and large pore volumes that provide numerous active sites on the catalyst surface [17,18,19,20]. In particular, Ru supported on activated carbon (Ru/C) was found to be highly effective for this reaction, achieving 100% LA conversion and 97.5% selectivity to GVL after 50 h at room temperature [21]. However, this catalyst also demonstrated rapid deactivation during the recycling process.

In an effort to increase the catalyst stability, mesoporous metal oxides, such as γ-Al_2_O_3_, SiO_2_, ZrO_2_, and TiO_2_ have emerged as promising alternatives to activated carbon owing to their high mechanical and thermal stability [22,23,24,25,26]. However, some of them show limited stability under acidic and hydrothermal conditions. Thus, SiO_2_ easily converts into a gel, while γ-Al_2_O_3_ has the tendency to transform into boehmite (AlO(OH)) at pH values between 4.5 and 11.5 [21,22]. On the other hand, TiO_2_ and ZrO_2_ have shown the highest stability in this reaction [23,24,25], despite the fact that Zr(OH)_4_ is generally more stable in water with respect to ZrO_2_ [26]. The superior activity of Ru/TiO_2_ was linked to the electronic properties of TiO_2_ which are favorable to the enhancement of metal support interactions and the formation of highly active Ru species in the selective hydrogenation of the carbonyl group [27,28,29,30,31].

Cyclodextrins (CDs) are water-soluble cyclic oligosaccharides composed of (α-1,4)-linked α-d-glucopyranose units presenting an internal hydrophobic cavity and a hydrophilic exterior surface. Alpha-, beta-, and gamma-CDs with respectively six, seven, and eight glucose units in the ring are the most common native cyclodextrins and among them, β-CD has received the greatest interest due to its high binding affinity and cost effectiveness [32,33]. Randomly methylated β-CD (RaMeβCD) is one of the most widely used β-CD derivatives with a degree of substitution (DS) of about 12.6 and a molecular structure that confers to these oligosaccharide surface-active properties and high solubility in water (>500 mg/mL). Although the possibility of utilizing native or modified cyclodextrins as structure-directing agents in the synthesis of silica nanostructures has been explored so far [34,35,36,37], a smaller number of reports have been devoted to the practical applications of these materials. Recently, we have developed an efficient CD-assisted colloidal approach to prepare hierarchically porous silica [37], as well as mesoporous alumina [38,39] and titania materials [40,41] with enhanced activity in photocatalysis [40,41,42], heterogeneous catalysis [43,44,45,46], and biocatalysis [47]. In particular, RuO_2_@TiO_2_ catalysts with tunable porosities, prepared using RaMeβCD-Pluronic P123 assemblies as a supramolecular template, gave excellent activity in the selective hydrogenation of methyl oleate to methyl stearate under mild reaction conditions [43]. 

Conventional TiO_2_ simple oxides still encounter limitations for application in heterogeneous catalysis due to the agglomeration of titania particles that generally imply inefficient mass transfer and poor pore accessibility, therefore declining the catalytic rate. Indeed, as pointed out by several authors, TiO_2_ undergoes sintering at high temperatures due to the coarsening and growth of metastable anatase and brookite crystallites, which further transform to rutile when a critical size is reached [48]. Such phase transformation is non-reversible due to the thermodynamic stability of the rutile phase which generally provokes a collapse of the nanostructure [49,50]. In an attempt to overcome this problem, the embedding of TiO_2_ particles in a porous and high surface area SiO_2_ matrix is an effective way to avoid particle aggregation. The uniform dispersion of titania particles in silica is nevertheless challenging due to the differences in hydrolysis and condensation rates of titanium and silicon alkoxides in water, which are identified as major limiting factors in the preparation of mixed TiO_2_-SiO_2_ mesoporous oxides.

In this study, a sophisticated CD-directed colloidal self-assembly strategy is demonstrated for the first time to tune the assembly process yielding uniform dispersion of TiO_2_ nanoparticles in a highly porous SiO_2_ matrix. Importantly, the porous matrix provides a confined environment not only to prevent TiO_2_ agglomeration, but also to control the growth process of Ru-catalyst yielding nanoparticles with different sizes and shapes (spherical, plate-like, lozenge-like, and rod-like). As a proof of concept, these catalysts were found to be particularly active in the aqueous phase hydrogenation of LA to GVL under mild reaction conditions, therefore opening new perspectives for the use of CD-based assemblies in the synthesis of high-performance catalysts.

## 2. Results

### 2.1. Method for the Preparation of the Catalysts

**Figure 1:**
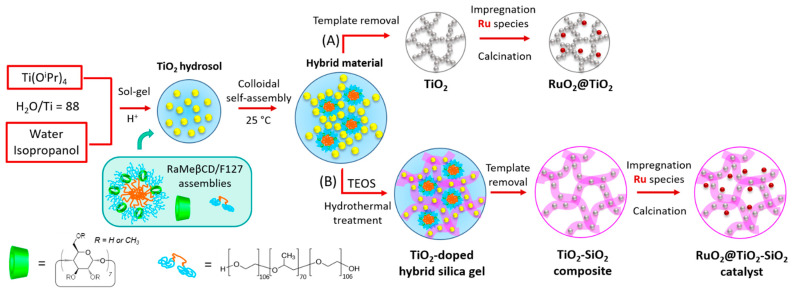
Schematic illustration of the colloidal approach for the preparation of the RuO_2_@TiO_2_ (**A**) and RuO_2_@TiO_2_-SiO_2_ (**B**) catalysts.

The mesoporous TiO_2_ and TiO_2_-SiO_2_ supported RuO_2_ catalysts were prepared according to the procedure depicted in Figure 1. First, a hybrid organic–inorganic material was obtained in aqueous phase by self-assembly of sol-gel synthesized TiO_2_ nanoparticles around the supramolecular assemblies formed between the randomly methylated β-CD (RaMeβCD) and the Pluronic F127. Then, the hybrid mesophase was either subjected to calcination at 500 °C to yield a mesoporous TiO_2_ material (Figure 1A) or further coated with a silica shell using the tetraethyl orthosilicate (TEOS) as a silica source (Figure 1B). By varying the TEOS concentration in the titania hydrosol, mesoporous titania-silica composites (TiO_2_-SiO_2_) with different silica loadings and porosities were obtained after template removal at 500 °C. In both syntheses, the obtained mesoporous supports were subsequently impregnated with an aqueous solution of ruthenium nitrosyl nitrate, followed by a thermal treatment at 400 °C under air flow, affording mesoporous RuO_2_@TiO_2_ and RuO_2_@TiO_2_-SiO_2_ heterostructures.

### 2.2. Characterization of the Supports

**Figure 2:**
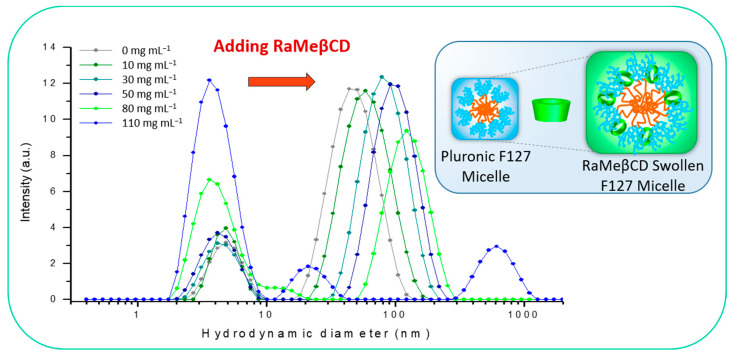
Dynamic light scattering (DLS) plots of the micellar solution of Pluronic F127 (10% *w*/*v*) prepared with increasing concentrations of RaMeβCD from 10 mg mL^−1^ to 110 mg mL^−1^.

To gain insight into the coassembly behavior of Pluronic F127 (PEO_106_PPO_70_PEO_106_) and RaMeβCD, DLS measurements were carried out at 25 °C. The Pluronic F127 concentrations used were above the critical micelle concentration (CMC) (0.7% *w/v* at 25 °C) [51]. Figure 2 shows the intensity size distributions of mixtures prepared with 10% *w/v* F127 and increasing amounts of RaMeβCD, from 10 to 110 mg mL^−1^. In the F127 solution prepared without RaMeβCD, the main scattering species are polymer oligomers and micelles with an average hydrodynamic diameter of 4.9 nm and 47 nm respectively. Upon addition of increasing amounts of RaMeβCD, the size of the micelles increases progressively from 59 nm (10 mg mL^−1^ RaMeβCD) to 98 nm (50 mg mL^−1^ RaMeβCD). Such a micellar growth may be explained by the ability of this cyclodextrin to locate preferentially at the interface layer of F127 micelles in interaction with the hydrophobic PPO blocks [38]. As a result of these interactions, the aggregation number of micelles increases, and interfaces tend to become more flexible inducing morphological changes when a certain packing density is reached. Hence, at 80 mg mL^−1^ RaMeβCD (RaMeβCD/F127 = 7.8), the decrease of the scattering intensity indicated the beginning of the restructuration of the micelles, which led to their total dissociation at 110 mg mL^−1^ RaMeβCD. Note that the micellar breakup occurred when the cyclodextrin was in large excess to the copolymer. Indeed, at 3% *w/v* F127, a 30 mg mL^−1^ RaMeβCD loading (RaMeβCD/F127 = 9.6) was sufficient to observe the micelle rupture, while at 15% *w/v* F127, micelles remained intact at a RaMeβCD loading as high as 110 mg mL^−1^ (RaMeβCD/F127 = 7.0) (Figure S1, ESI). Typically, the restructuration of interfaces was found to occur for RaMeβCD/F127 molar ratios higher than 7. Based on these results, the template chosen for the preparation of the supported catalysts was the mixture composed of 10% *w/v* Pluronic F127 and 50 mg mL^−1^ RaMeβCD (RaMeβCD/F127 = 4.8), as in this concentration range micelles maintained their structural integrity.

**Figure 3:**
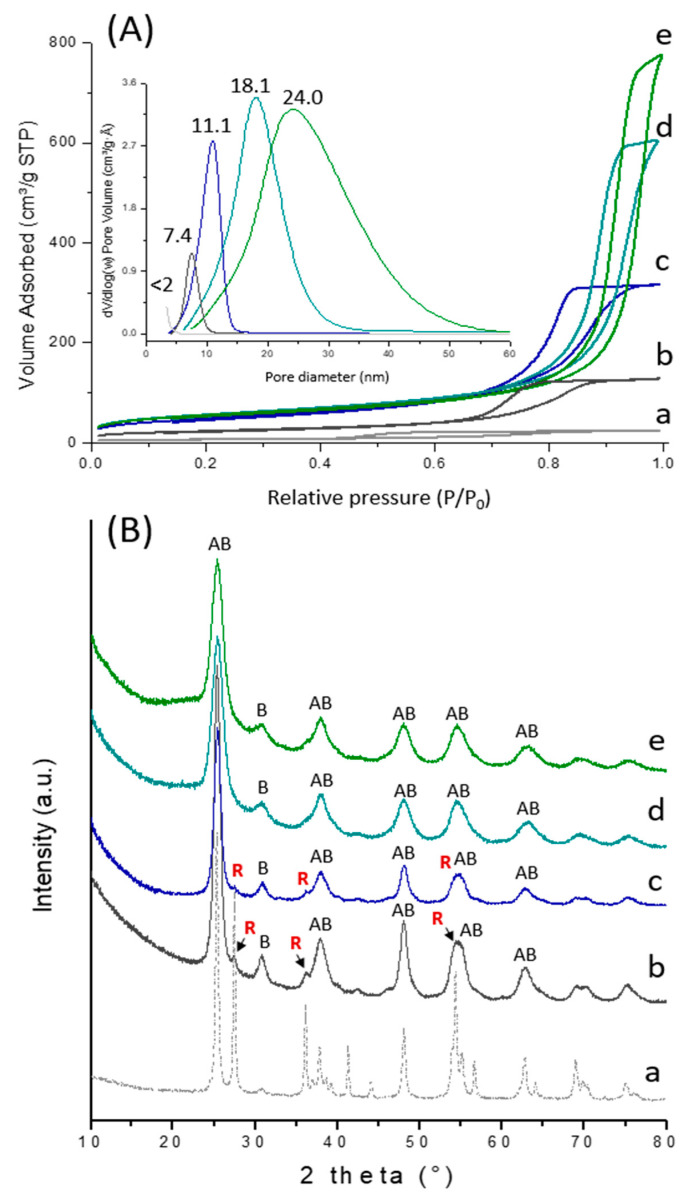
N_2_-adsorption isotherms and corresponding pore size distributions (inset) (**A**) and XRD patterns (**B**) of TiO_2_-sg (a), TiO_2_-ns (b), TiO_2_-SiO_2_ 3:1-ns (c), TiO_2_-SiO_2_ 3:2-ns (d), TiO_2_-SiO_2_ 3:3-ns (e). The “A”, “B” and “R” denote the anatase, brookite and rutile phases respectively.

Figure 3A shows the N_2_-adsorption isotherms and corresponding pore size distributions (inset) of the TiO_2_ material and TiO_2_-SiO_2_ composites prepared using the colloidal approach (Figure 1). All solids displayed a type IV isotherm with a type H1 hysteresis loop characteristic of mesoporous materials. The adsorption isotherm of the template-free sol-gel TiO_2_ (TiO_2_-sg) showed a capillary condensation step that started at a relative pressure (P/P_0_) of about 0.45, consistent with the formation of compact microstructures (0.03 cm^3^ g^−1^ pore volume and less than 2 nm diameter). Upon addition of the RaMeβCD-Pluronic F127 assemblies, a steep rise in the N_2_ uptake occurred at P/P_0_ > 0.7 indicating the formation of larger mesopores with 7.4 nm pore diameters. Moreover, the increase in both surface area (from 21 to 80 m^2^ g^−1^) and pore volume (from 0.03 to 0.20 cm^3^ g^−1^) (Table 1) is a signature of the template effect, as evidenced also in the case of bare silica (Figure S2, Table S1, ESI). Interestingly, the textural characteristics were further improved through sol-gel polymerization of the TEOS around the network formed by the conjunction of RaMeβCD-Pluronic F127 template and TiO_2_ nanoparticles. Indeed, by varying the composition of silica in the composite material, it was possible to tune the average pore diameter in a broad range between 10.9 nm and 20.4 nm (Table 1). 

X-ray diffraction (XRD) patterns also confirmed that both the supramolecular template and silica loading had an impact on the crystalline properties of titania-based solids (Figure 3B). Indeed, in the TiO_2_-sg material, the intense sharp peak at 2θ = 27.4° is characteristic of the (110) plane of the rutile polymorph (R) (JCPDS card no. 00-034-0180), which results from the transformation of metastable anatase (A) (JCPDS card no. 00-021-1272) and brookite (B) (JCPDS card no. 01-076-1934) during calcination. The crystallite sizes of those polymorphs determined from the Scherrer formula were 36 nm (A), 19 nm (B), and 60 nm (R). The rapid growth of rutile crystallites may be attributed to the high level of aggregation of particles in the sol-gel TiO_2_ material, facilitating the contact between neighboring grains. Indeed, as shown previously by Zhang et al. [52,53], when the anatase particles are in intimate contact with each other, the interfaces of the contacting grains provide the nucleation sites of the rutile phase. Once the nucleation is initiated, rutile particles grow very fast and the phase transformation spreads quickly throughout the whole material. Noticeably, when the RaMeβCD-Pluronic F127 assemblies were used as a template, the intensities of rutile reflections were markedly attenuated, and they totally disappeared when silica was incorporated to the titania network. Moreover, the broadening of the XRD peaks is consistent with the formation of smaller particles resulting from the delay in phase transformation. Thus, the size of crystallites determined from the Scherer formula decreased from 36.4 nm (A) and 18.7 nm (B) for TiO_2_-sg to 11.0 nm (A) and 4.5 nm (B) for TiO_2_-ns and further to 6.1 (A) and 2.7 nm (B) for TiO_2_-SiO_2_ 3:3-ns. Such a decrease in the size of crystallites also explains the increase in the specific surface area and pore volume of TiO_2_-SiO_2_ composites (Table 1).

Taken together, these data indicate that both the RaMeβCD-F127 assemblies and the silica network play a key role in inhibiting crystal growth and stabilizing anatase and brookite polymorphs with respect to the rutile. 

### 2.3. Characterization of the Supported Catalysts

**Figure 4:**
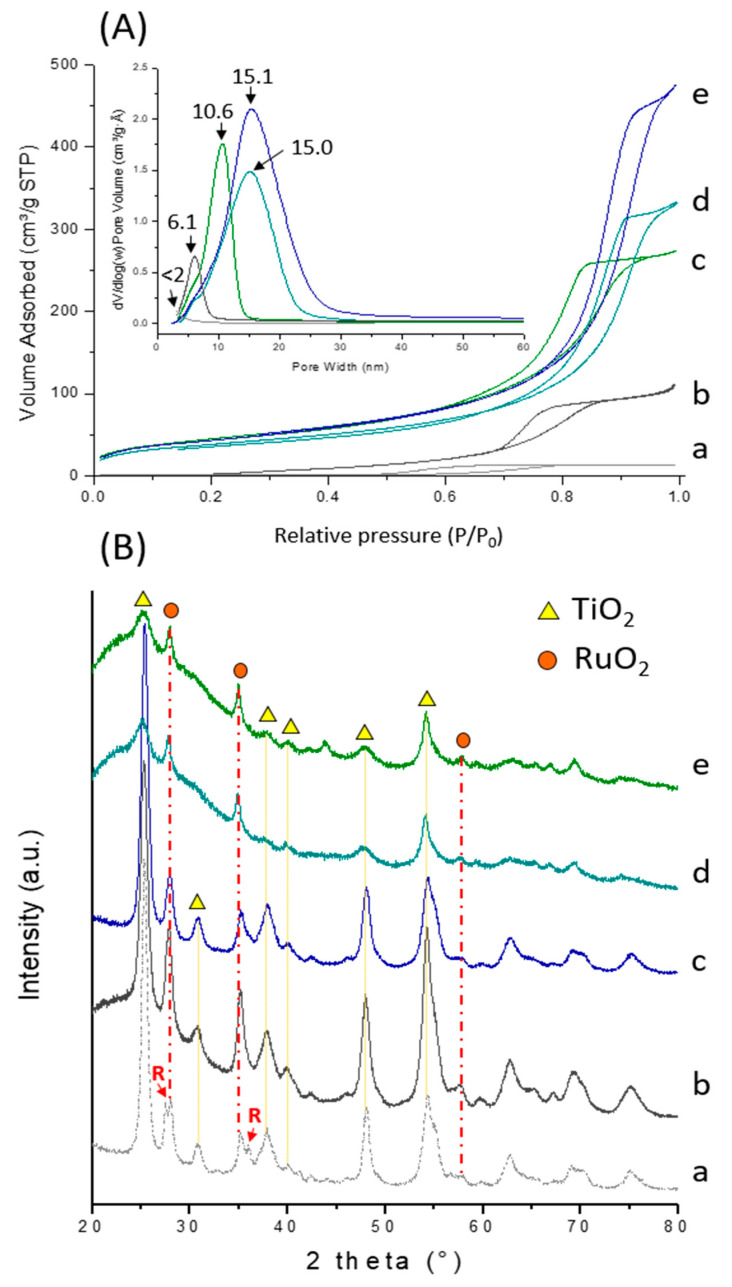
N_2_ adsorption isotherms and corresponding pore size distributions (inset) (**A**) and XRD patterns (**B**) of RuO_2_@TiO_2_-sg (a), RuO_2_@TiO_2_-ns (b), RuO_2_@TiO_2_-SiO_2_ 3:1-ns (c), RuO_2_@TiO_2_-SiO_2_ 3:2-ns (d), and RuO_2_@TiO_2_-SiO_2_ 3:3-ns (e). “R” denotes the rutile phase.

To further investigate the impact of RuO_2_ particles on the textural and structural characteristics of supported catalysts, N_2_-adsorption and XRD analyses were carried out. From the N_2_-adsorption isotherms (Figure 4A), it can be seen that all TiO_2_-based materials retained their mesoporous structure after dispersion of the active elements. As a general trend, the specific surface area, pore volume and pore size decreased slightly after dispersion of RuO_2_ particles while the pore architecture was not altered, which could be beneficial for the diffusion of reactants and products during the catalytic process. For instance, the surface area, pore volume and pore diameter were determined to be 156 m^2^/g, 0.72 cm^3^/g and 15.1 nm respectively for the RuO_2_@TiO_2_-SiO_2_ 3:3-ns catalyst (Table 2) and 193 m^2^/g, 1.17 cm^3^/g and 24.0 nm respectively for the corresponding TiO_2_-SiO_2_ 3:3-ns support (Table 1). It is noteworthy to mention that all those catalysts had much better textural characteristics compared to other crystalline TiO_2_-supported Ru catalysts reported in the literature [26] due to the small-sized TiO_2_ particles resulting from the high hydrolysis ratio employed under our synthesis conditions (H_2_O/Ti = 88). 

Compared to bare TiO_2_ and TiO_2_-SiO_2_ supports, the XRD patterns of supported catalysts (Figure 4B) revealed the existence of two additional reflection lines at 2θ = 27.8° and 34.9° originating from the (110) and (101) planes respectively of the tetragonal RuO_2_ (JCPDS 00-043-1027). Note that among the five catalysts, RuO_2_@TiO_2_-sg showed the highest RuO_2_ reflection intensities indicating larger particle sizes with an average diameter of 15.8 nm, as determined from the line broadening of the (110) reflection. This result is consistent with the epitaxial growth of RuO_2_ phase over rutile-TiO_2_, which is the predominant polymorph in the TiO_2_-sg support. Indeed, the (110) and (101) planes of RuO_2_ overlap with the (110) and (101) planes of rutile-TiO_2_ due to the structural matching between these two phases, both of which share a tetragonal lattice symmetry and have very similar lattice parameters (a = 4.59 Å, c = 2.96 Å for rutile-TiO_2_ and a = 4.49 Å, c = 3.10 Å for RuO_2_) [54]. Similarly to the supramolecular template, the sol-gel polymerization of TEOS around TiO_2_ particles permitted stabilization of anatase and brookite with respect to rutile. RuO_2_ particles dispersed over this support were well-crystallized, as confirmed by the sharp peaks originating from the reflection planes of tetragonal RuO_2_ (Figure S3, ESI). On the other hand, the combined use of the supramolecular RaMeβCD-Pluronic F127 template and TEOS resulted in a further broadening of the reflection peaks of RuO_2_ and a reduction in the size of RuO_2_ crystallites which decreased from 15.8 nm (RuO_2_@TiO_2_-sg) to 9.9 nm (RuO_2_@TiO_2_-ns) and to 9.0 nm (RuO_2_@TiO_2_-SiO_2_ 3:1-ns) (Table 2).

**Figure 5:**
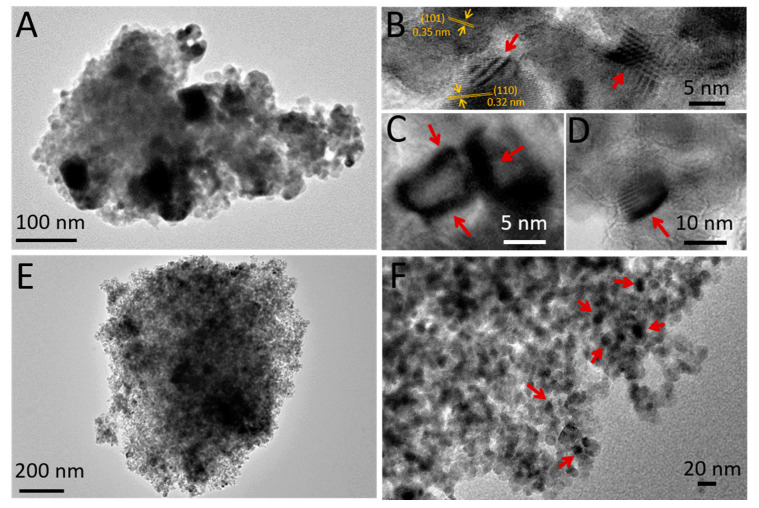
Representative TEM images of Ru@TiO_2_-sg (**A**–**D**) and Ru@TiO_2_-ns (**E**,**F**). Red arrows indicate the epitaxial growth of RuO_2_ crystallites over rutile-TiO_2_-sg (**B**–**D**) and formation of Ru clusters with an average diameter of 15–20 nm over TiO_2_-ns (**F**).

Transmission electron microscopy (TEM) images confirmed that the Ru@TiO_2_-sg catalyst was composed of large RuO_2_ agglomerates with irregular sizes varying from 20 to 50 nm in diameter (dark regions) dispersed in a fully dense titania microstructure (white regions) (Figure 5A). Titania particles showed lattice fringes with 0.354 nm and 0.326 nm interlayer distances corresponding to anatase (101) and rutile (110) planes respectively, in line with XRD results (Figure 5B). Moreover, elongated heterogeneous structures were also identified (red arrows), consistent with the epitaxial growth of RuO_2_ crystallites over rutile-TiO_2_ (Figure 5C,D). Importantly, the nanostructured RaMeβCD-templated TiO_2_-ns support provided a more uniform dispersion of the active phase, indicating formation of Ru clusters with an average diameter of 15–20 nm located either on the external surface of titania particles or encapsulated inside the pores (Figure 5E,F). The numerous void spaces observed between the TiO_2_ particles (white regions) confirm the important role played by the RaMeβCD-swollen F127 micelles in the pore expansion, in agreement with N_2_-adsorption results.

High-angle annular bright-field and dark-field scanning transmission electron microscopy (HAABF- and HAADF-STEM, respectively) images together with EDS mappings were further used to examine the distribution of Ti, Si, Ru, and O elements in the three RuO_2_@TiO_2_-SiO_2_-ns catalysts. Note that the bright particles in the HAADF-STEM images are those that contain the heaviest element, i.e., Ru.

**Figure 6:**
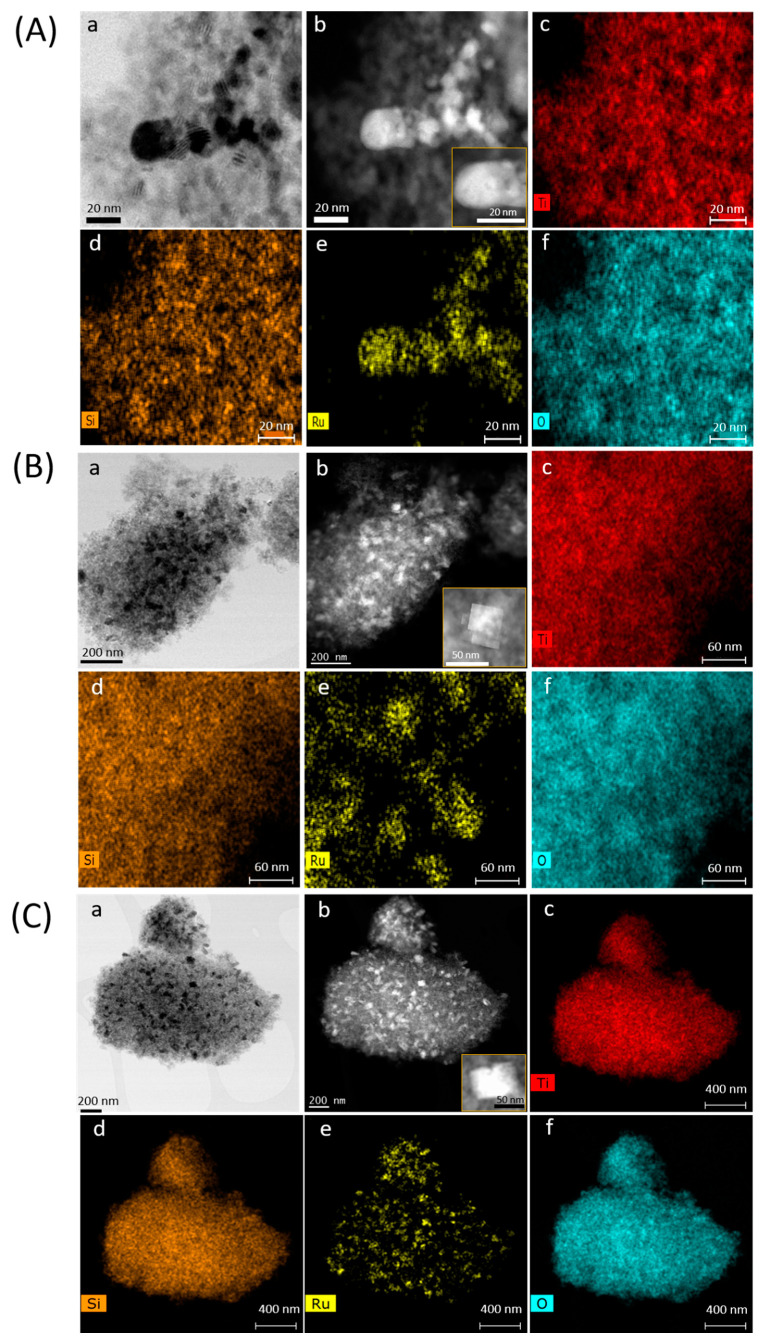
HAABF- (a) and HAADF-STEM (b) images together with corresponding energy dispersive X-ray (EDX) elemental mapping of Ti (c), Si (d), Ru (e), and O (f) for RuO_2_@TiO_2_-SiO_2_ 3:1-ns (**A**), RuO_2_@TiO_2_-SiO_2_ 3:2-ns (**B**), and RuO_2_@TiO_2_-SiO_2_ 3:3-ns (**C**) catalysts. Insets in subfigures b show a higher magnification of RuO_2_ particles.

As shown in Figure 6, the Ti, Si, and O elements were distributed evenly throughout the selected area of all catalysts. However, the growth of RuO_2_ nanostructures was different depending on the amount of silica incorporated within the titania network. Indeed, the RuO_2_@TiO_2_-SiO_2_ 3:1-ns catalyst prepared with the lowest silica loadings provided spherical particles that tended to agglomerate into grapes (Figure 6A and Figure S4, ESI). A closer examination by HR-TEM revealed that Ru nanostructures were composed of globular Ru clusters with 5 nm diameter agglomerated into larger particles with an average size of 20–30 nm (Figure S5, ESI). Importantly, the addition of increasing amounts of silica to the titania network (TiO_2_-SiO_2_ 3:2-ns and TiO_2_-SiO_2_ 3:3-ns solids) involved a more uniform dispersion of Ru particles as well as a shape transformation from circular particles to heterogeneous structures composed of plate-like or lozenge-shaped crystals having an average particle size of 40–50 nm (Figure 6B,C and Figure S7, ESI). Moreover, in some local regions, it can be seen that RuO_2_ clusters tend to further aggregate, forming rod-like particles of 50–100 nm length and 20–40 nm width (Figure S6, ESI).

**Figure 7:**
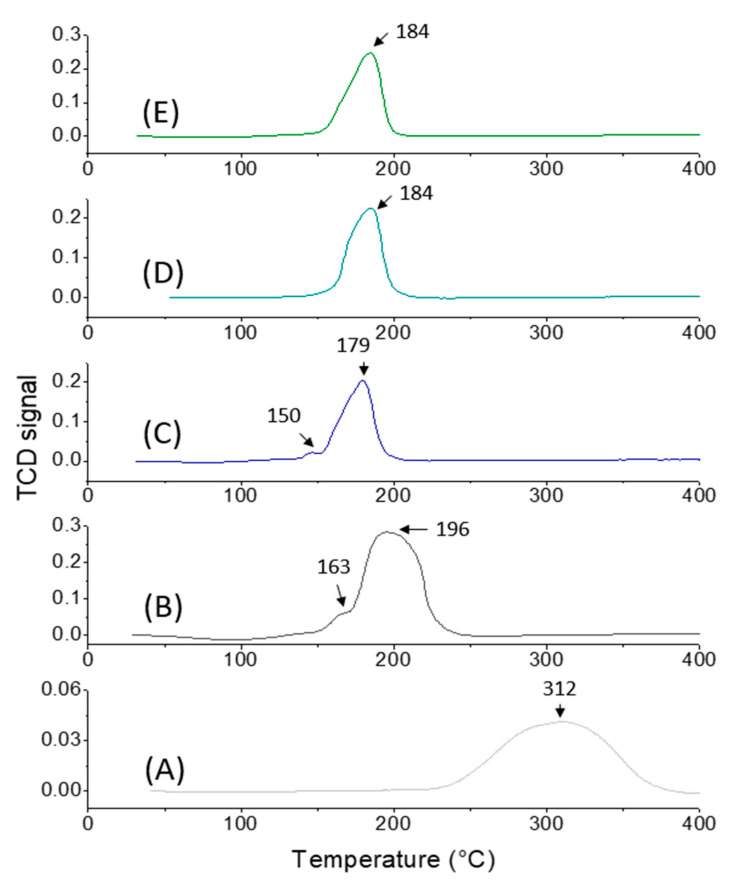
Temperature programmed reduction (TPR) patterns of RuO_2_@TiO_2_-sg (**A**), RuO_2_@TiO_2_-ns (**B**), RuO_2_@TiO_2_-SiO_2_ 3:1-ns (**C**), RuO_2_@TiO_2_-SiO_2_ 3:2-ns (**D**), and RuO_2_@TiO_2_-SiO_2_ 3:3-ns (**E**).

Temperature programmed reduction (TPR) measurements further illustrate the different reducibility behavior of RuO_2_ on different supports (Figure 7). Thus, RuO_2_@TiO_2_-sg presented a broad peak beginning at 230 °C and extending up to 380 °C assigned to the reduction of Ru^4+^ to Ru^0^ (Figure 7A). Such a high reduction temperature is likely attributable to the formation of epitaxial layers of RuO_2_ in strong interaction with rutile-TiO_2_, in line with the XRD and TEM results. A clear shift of this temperature to lower values was observed on the RuO_2_@TiO_2_-ns catalyst, which displayed two detached peaks suggesting formation of two different types of Ru species (Figure 7B) [43]. The first peak, centered at 163 °C, may be related to the reduction of surface particles that interact weakly with titania surfaces and the second one, centered at 196 °C extending up to 240 °C, may be attributed to the reduction of RuO_2_ particles incorporated within the pores. On the other hand, RuO_2_ particles supported over mixed oxides globally displayed lower reduction temperatures in the range of 180–185 °C which did not exceed 200 °C (Figure 7C–E). Moreover, as the silica content increased, the material became more porous and the shoulder on the primary reduction peak disappeared, suggesting the formation of one type of particle incorporated within the pores. It is worthy to note that the RuO_2_@TiO_2_-SiO_2_ 3:3-ns catalyst displayed a lower reduction temperature and narrower peak compared to the RuO_2_@TiO_2_-SiO_2_ 3:3-sg catalyst (Figure S8, ESI) indicating higher homogeneity and readily reducible RuO_2_ particles. Notably, no significant reduction of the support was observed when the temperature was raised to 800 °C (Figure S8, ESI).

### 2.4. Catalytic Tests

The catalytic performances of supported catalysts were investigated in the hydrogenation of levulinic acid (LA) to γ-valerolactone (GVL). Prior to the catalytic reaction, RuO_2_ was reduced in metallic Ru using a 5% H_2_/Ar flow at 400 °C for 2 h, and then immediately transferred in autoclave. All experiments were performed at 50 °C under a hydrogen pressure of 50 bar.

**Figure 8:**
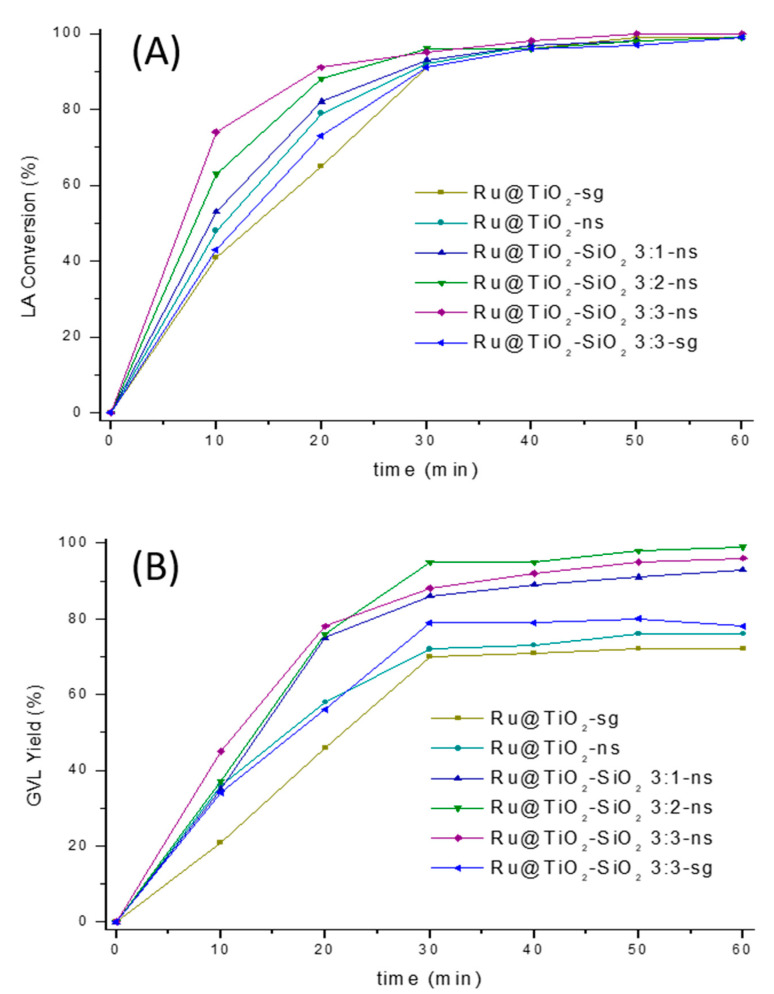
The influence of reaction time on the LA conversion (**A**) and the GVL yield (**B**) by various catalysts. Reaction conditions: 0.66 g LA (5.68 mmol), 77.6 mg supported catalyst (0.019 mmol Ru), 50 bar H_2_, 20 mL H_2_O, temperature 323 K, and reaction time 1 h.

The efficient catalytic conversion of levulinic acid to γ-valerolactone in aqueous phase involves two main steps: first the hydrogenation of LA, yielding 4-hydroxypentanoic acid (4-HPA) as an intermediate, followed by the subsequent cyclization of the latter forming GVL (see Table 3) [26,55,56,57,58]. Figure 8 shows concentration versus time profiles for LA and GVL obtained with the different catalysts during the first 60 min of reaction. Ru deposited on TiO_2_-SiO_2_ 3:3-sg composite is added for comparison. At 2.5 wt.% Ru loading, all catalysts provided a sufficient amount of active sites to initiate the hydrogenation reaction and were able to give near quantitative LA conversions with GVL yields comprised between 72% and 99%. In all reaction mixtures, the only intermediate product detected was 4-HPA (see ^1^H NMR spectra Figures S9 and S10, ESI) in agreement with literature data for reactions performed in water [13,15,59]. Other intermediates such as α-angelicalactone or GVL hydrogenation products (i.e., 1,4-pentanediol or 2-methyltetrahydrofuran) were not observed under our reaction conditions, indicating selectivity towards GVL formation.

It is interesting to note that both LA conversion and GVL yield were influenced by the nature of the support material. For instance, the highest conversions were achieved with Ru particles supported on mixed TiO_2_-SiO_2_ oxides prepared by the template approach, intermediate activities were found for catalysts supported on nanostructured TiO_2_, and the lowest LA conversions were obtained with Ru dispersed over TiO_2_-sg and TiO_2_-SiO_2_-sg supports. A similar tendency was observed also in the GVL yield which increased with silica loading, reaching a maximum of 99% for the Ru@TiO_2_-SiO_2_ 3:2-ns catalyst.

To evaluate the efficiency of different catalysts, activities were then compared after 20 min of reaction time. A compilation of the data is given in Table 3. As expected, the blank TiO_2_ and TiO_2_-SiO_2_ supports exhibited insignificant catalytic activity due to the absence of active sites (entries 1 and 2). The Ru@TiO_2_-sg catalyst gave 65% LA conversion and 46% GVL yield with a productivity of 4.0 mol_GVL_ g^−1^_metal_ h^−1^ (entry 3). Notably, when the CD-based assemblies were used as template, the GVL yield increased to 58%, affording a productivity of 5.1 mol_GVL_ g^−1^_metal_ h^−1^ (entry 4). Interestingly, further improvement in the catalytic performance was noticed upon increasing the silica loading. Indeed, LA conversions obtained with the three Ru@TiO_2_-SiO_2_-ns catalysts were higher than 80% and GVL yields were between 75% and 78%, giving productivities in the range of 6.59–6.86 mol_GVL_ g^−1^_metal_ h^−1^ (entries 5–7). Note that the best results were obtained with Ru@TiO_2_-SiO_2_ 3:3-ns, affording 91% LA conversion and 78% GVL yield with a productivity of 6.85 mol_GVL_ g^−1^_metal_ h^−1^, which is almost 1.65-fold higher than the results obtained with the Ru@TiO_2_-sg catalyst. For comparison, Ru@TiO_2_-SiO_2_ 3:3-sg prepared by conventional sol-gel process gave intermediate activities between Ru@TiO_2_-sg and Ru@TiO_2_-ns, with a GVL yield of 56% and a productivity of 4.9 mol_GVL_ g^−1^_metal_ h^−1^ (entry 8). In comparison, commercial 5%Ru@C gave an almost full LA conversion in the first 20 min, but it produced only 66% GVL yield.

Our results can be compared with those reported by Almeida et al. [60] who obtained 96% LA conversion and 89% GVL yield with a 5%Ru@TiO_2_-SiO_2_ catalyst after 6 h at 100 °C and 20 atm H_2_. However, in this study, the catalytic tests were performed in ethanol, where ethyl levulinate is expected to form as a byproduct through the transesterification of levulinic acid [61]. No data were available however for the reaction performed in water. Using a 5%Cu/γ-Al_2_O_3_ catalyst, 87% yield of GVL was reached by Putrakumar et al. [62] in gas phase at 265 °C and 30 mL H_2_ flow. The catalyst showed good activity during the initial hours of reaction, but tended to deactivate as the reaction time with increased. Jones et al. [63] achieved 90% yield in GVL at 100 °C under 5 bar H_2_ with a 1%Ru/C catalyst stabilized by a small amount of PVA (PVA/Ru = 0.1). However, during the recycling experiments, this catalyst showed a decline in activity which was attributed to the removal of PVA from the Ru surface, followed by particle sintering. In another study, Piskun et al. [56] obtained 86% LA conversion and 79% GVL yield with a 1%Ru@TiO_2_ catalyst in water after 4 h at 90 °C and 45 bar H_2_. As already well-established in the literature, water strongly accelerates the reaction rate with respect to organic solvents as it participates to the hydrogenation of the C=O group of LA by decreasing the energy barrier [13,24]. Moreover, although H_2_ is less soluble in water than in organic solvents, the ability of water to promote the hydrogen spillover by transporting H atoms on the catalyst surface appears also to contribute to its enhanced activity [16,64,65].

Overall, our results indicate that both the supramolecular RaMeβCD/F127 assemblies and the silica network have a beneficial effect on the catalytic properties of Ru-based composites. Indeed, comparison of the activities obtained with Ru@TiO_2_-ns (entry 4) and Ru@TiO_2_-sg (entry 3), as well as with Ru@TiO_2_-SiO_2_ 3:3-ns (entry 7) and Ru@TiO_2_-SiO_2_ 3:3-sg (entry 8), clearly reveal the determinant role of the supramolecular template on the catalytic performance of Ru. The higher performance of those catalysts can be largely attributed to a combined effect of improved structural and textural properties of the support and more uniform dispersion of the Ru particles, as confirmed by our XRD, N_2_-adsorption, and HR-TEM and HAADF-STEM results. Moreover, the results obtained with mixed oxides also suggest that there is a significant potential for further activity improvement by embedding titania particles within the silica matrix prior to Ru dispersion. The silica network seems to play a key role not only on the characteristics of the support itself, whose pore diameter increased from 7.4 to 24.0 nm, but also on the morphology of Ru particles which transformed from spherical to plate-like, lozenge-like, and rod-like nanocrystals with increased silica loading. Although the thickness of those structures could not be determined from our TEM images, the evolution observed on particle morphology may also have some effects on the catalytic activity through orientation of certain facets of Ru nanocrystals. Indeed, DFT calculations have suggested that Ru nanocrystals with exposed {111} facets were more effective than Ru nanoparticles in catalyzing nitrogen reduction for ammonia synthesis [66] and have recently shown very beneficial effects on the catalytic activity towards oxygen evolution reactions [67]. 

To gain a deeper understanding on the role of the chemical nature of the support material, we also conducted a set of control experiments on other mixed oxides with high surface areas and pore volumes (Table S2, ESI). Results revealed that the catalytic activity of sol-gel and nanostructured Ru@TiO_2_-SiO_2_ 3:3 catalyst was superior to that of the Ru@TiO_2_-Al_2_O_3_ 3:3 and Ru@SiO_2_-Al_2_O_3_ 3:3 catalysts prepared by a similar colloidal approach using boehmite AlO(OH) particles as precursor of γ-Al_2_O_3_ [38]. Although SiO_2_-Al_2_O_3_ supports presented the best textural characteristics among the three mixed oxides, LA conversions and GVL yields obtained under the same reaction conditions (50 °C, 50 bar H_2_, 20 min) were found to evolve in the order Ru@TiO_2_-SiO_2_ 3:3 > Ru@TiO_2_-Al_2_O_3_ 3:3 > Ru@SiO_2_-Al_2_O_3_ 3:3 (Figure S11, ESI). This means, nonetheless, that the surface areas and pore volumes are not the only factors that explain the marked improvement in the catalytic performance of Ru@TiO_2_-SiO_2_. Additionally, the Lewis acid sites in mixed SiO_2_-TiO_2_ composites [68] may also contribute to the enhanced performance of our Ru catalysts. Indeed, Kumar et al. [69,70], found that Lewis acid sites were favorable to the conversion of LA to GVL, while Brønsted acid sites, which are normally absent in the structure of calcined TiO_2_-SiO_2_ composites [71,72], were responsible for the GVL ring opening to valeric acid (VA). It is worth noting that all these factors are interlinked and a harmonization between them is necessary to achieve a high performance catalyst for selective hydrogenation of LA to GVL. The more adequate electronic and crystal properties of the semi-conductor may also be favorable to the improvement of metal support interactions and the stability of the catalyst in water, both of which are likely to contribute to the superior activity of Ru@TiO_2_-SiO_2_ [31].

**Figure 9:**
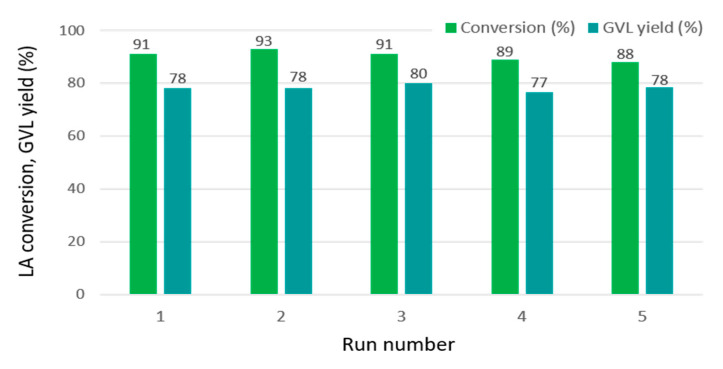
Reusability tests on the Ru@TiO_2_-SiO_2_ 3:3-ns catalyst. Reaction conditions: 0.66 g LA (5.68 mmol), 77.6 mg supported catalyst (0.019 mmol Ru), 50 bar H_2_, 20 mL H_2_O, temperature 323 K, and reaction time 20 min.

Recovery and recycling of the catalyst is an important challenge for industrial applications, especially when corrosive reactants such as LA are used, which may cause metal leaching and deactivation of the catalyst. Although Ru/C has proved to be one of the most active catalysts in the aqueous phase hydrogenation of LA, Al-Shaal et al. [21] have noticed a rapid deactivation during the recycling process. As illustrated in Figure 9, our best catalyst Ru@TiO_2_-SiO_2_ 3:3-ns was very stable in this reaction and could be reused in at least five consecutive catalytic runs without significant loss of catalytic performance. Its robustness can be ascribed to the combined effect of uniform and stable dispersion of Ru nanocrystals in a pore-confined space and their enhanced interactions with the TiO_2_-SiO_2_ support, as revealed by N_2_-adsorption, HR-TEM, HAADF-STEM, and TPR.

## 3. Materials and Methods

### 3.1. Chemicals

Randomly methylated β-cyclodextrin (denoted RaMeβCD, average degree of molar substitution (DS) 1.8 and average molar weight (Mw) 1310 g mol^−1^) was a gift from Wacker Chemie GmbH. Pluronic F127 with a chemical formula PEO_106_PPO_70_PEO_106_ (PEO = poly(ethylene oxide) and PPO = poly(propylene oxide)) (Mw 12,600 *g* mol^−1^), titanium isopropoxyde (Ti(O^i^Pr)_4_) (Mw 284.3 g mol^−1^, d 0.96 g cm^−3^), tetraethylorthosilicate (TEOS) (Mw 208.33 g mol^−1^, d 0.93 g cm^−3^), nitric acid (HNO_3_, 68%), levulinic acid (LA) (Mw 116.11 g mol^−1^), and 4-hydroxypentanoic acid (4-HPA) (Mw 118.13 g mol^−1^) were purchased from Sigma Aldrich. Ruthenium (III) nitrosyl nitrate (Ru(NO)(NO_3_)_3_) (1.5% Ru, Mw 318.10 g mol^−1^) was procured from STREM Chemicals. All chemicals were used as received without further purification.

### 3.2. Preparation of Mesoporous TiO_2_ and SiO_2_-TiO_2_ Materials

The titanium dioxide (TiO_2_) hydrosol was synthesized according to a previously reported sol-gel method [73]. Briefly, to a mixture of 30 mL (0.1 mol) Ti(O^i^Pr)_4_ and 27 mL (0.35 mol) isopropanol, 160 mL of hot distilled water was added rapidly at 85 °C under vigorous stirring (hydrolysis ratio h = H_2_O/Ti = 88). After 15 min, 1.3 mL of nitric acid (HNO_3_/Ti = 0.2) was added dropwise to peptize the viscous precipitate and the mixture was maintained under reflux at 85 °C for 16 h. A stable translucent suspension of TiO_2_ nanoparticles (pH 2.1) crystallized in anatase (~70%) and brookite (~30%) was recovered at the end of the synthesis. The titanium concentration in the sol was 0.48 mol L^−1^, as determined by weight loss on ignition at 1000 °C for 2 h. For the preparation of nanostructured TiO_2_, Pluronic F127 (10 wt.%, F127/Ti molar ratio = 0.0165) and RaMeβCD (50 mg mL^−1^, RaMeβCD/Ti molar ratio = 0.079) were added successively to 100 mL of the above titania sol. The mixture was stirred at room temperature for 3 h, and then allowed to equilibrate for 24 h. A nanostructured porous titania-denoted TiO_2_-ns was obtained after drying the sol at 60 °C for 72 h, followed by rinsing with ethanol and further calcination at 500 °C for 2 h using a heating ramp of 5 °C min^−1^. For the preparation of nanostructured TiO_2_-SiO_2_ composites, 0.648 g TEOS (Ti/Si molar ratio = 3) was added to 20 mL of the hybrid RaMeβCD-F127@TiO_2_ suspension and maintained under stirring at room temperature for 1 h. Subsequently, the mixture was placed in an autoclave for hydrothermal treatment at 150 °C for 48 h. The recovered gel was placed in a Soxhlet extractor and rinsed with HCl (4%)-ethanol solution to remove the organic template. Finally, the solid was dried at 100 °C for 16 h, then calcined in air at 500 °C for additional 2 h. This sample was denoted TiO_2_@SiO_2_ 3:1-ns. TiO_2_@SiO_2_ 3:2-ns and TiO_2_@SiO_2_ 3:3-ns were prepared under the same conditions using 1.297 g and 1.945 g TEOS, respectively. In some experiments, template-free sol-gel titania (TiO_2_-sg) and titanosilicate (TiO_2_-SiO_2_-sg) were also prepared for comparison.

### 3.3. Preparation of RuO_2_@TiO_2_ and RuO_2_@TiO_2_-SiO_2_ Catalysts

Supported catalysts were prepared by an impregnation method using Ru(NO)(NO_3_)_3_ as precursor of RuO_2_ because it was previously shown to provide the best results in terms of turn-over frequencies [26]. Typically, 800 mg of calcined TiO_2_ or TiO_2_-SiO_2_ material were impregnated with 6 mL of an aqueous solution of Ru(NO)(NO_3_)_3_ (0.033 mol L^−1^ Ru). The suspension was maintained under stirring at 75 °C until the water was completely evaporated. Then, this solid was calcined at 400 °C for 4 h under air flow. The resulting catalysts were denoted Ru@TiO_2_-ns(sg) or Ru@TiO_2_-SiO_2_-x:y-ns(sg), where x:y indicates the Ti/Si molar ratio, whereas ns and sg refer to nanostructured and sol-gel catalysts, respectively. 

### 3.4. Characterization Methods

Dynamic light scattering (DLS) measurements were performed at 25 °C with a Malvern Zeta Nanosizer instrument equipped with a 4 mW He-Ne laser operating at 633 nm using a backscattering detection system. The scattering angle θ was 173°. Powder X-ray diffraction patterns were acquired using a Siemens D5000 X-ray diffractometer in a Bragg–Brentano configuration with a Cu Kα radiation source. Scans were run over the angular 10° < 2θ < 80° domain with a step size of 0.02° and a step time of 2 s. Crystalline phases were identified based on the JCPDS database. FullProf software [74] and its graphical interface WinPlotr [75] were used for the refinement of the diffractograms. Profile matching refinement was used to determine the unit cell parameters of TiO_2_, background, peak shape, and zero shift. The quality of the fit was determined visually by inspection of the difference plot and statistically by the goodness of fit (χ^2^), defined by:

(1) $$\chi^{2}=\;\sum w_{i}{\left(y_{io}-y_{ic}\right)}^{2}/\left(N-P\right)\;$$

where *w_i_* is the weight assigned to each observation; *y_io_* and *y_ic_* are the observed and calculated intensities, respectively, at the *i*th step; *N* is the number of points used in the refinement; and *P* is the number of least-squares parameters refined. The refinement was considered satisfactory when χ^2^ was less than 3. Typical results obtained from profile matching on anatase/brookite and anatase/brookite/rutile mixtures are shown in Figure S12 (ESI). The average size (D) of the TiO_2_ crystallites was then determined using the Scherrer formula [76], D = Kλ/(β cos θ), where K is the shape factor (a value of 0.9 was taken in this study assuming that particles are spherical), λ is the X-ray radiation wavelength (1.54056 Å for Cu Kα), β is the full width at half-maximum (fwhm), and θ is the Bragg angle. The average size of the RuO_2_ crystallites was estimated from the line broadening of the (110) reflection. N_2_ adsorption–desorption isotherms were collected at −196 °C using an adsorption analyzer, Micromeritics Tristar 3020. Prior to analysis, 100–150 mg samples were outgassed at 260 °C overnight to remove the species adsorbed on the surface. From N_2_ adsorption isotherms, specific surface areas were evaluated by the Brunauer-Emmett-Teller (BET) method [77] and pore size distributions were determined using the Barrett, Joyner, and Halenda (BJH) method assuming a cylindrical pore structure. Relative errors were estimated to be 5% for the surface area, 5% for the pore volume, and 20% for the BJH pore diameter. Temperature programmed reduction (TPR) measurements were carried out on a Micromeritics AutoChem 2920 chemisorption analyzer equipped with a thermal conductivity detector (TCD) used to monitor the H_2_ consumption. Prior to analysis, 30–40 mg samples were placed in a U-tube quartz reactor and outgassed under argon flow at 120 °C for 2 h. After cooling to 25 °C, samples were reheated between 30 °C and 400 °C in a 5% H_2_/Ar (*v*/*v*) flow with a flow rate of 10 mL min^−1^ and a heating rate of 10 °C min^−1^. Transmission electron microscopy (TEM), high-resolution TEM (HR-TEM), high-angle annular dark-field and bright-field scanning TEM (HAABF- and HAADF-STEM, respectively) images, and energy dispersive X-ray spectrometry (EDS) elemental mapping results were recorded on a MET FEI TITAN Themis 300 microscope with an accelerating voltage of 300 kV. The resolution of the STEM was less than 70 pm. The instrument is equipped with a Super-X EDX detector system with superior sensitivity and a fast EDS mapping. Four silicon drift detectors (SDDs) are integrated in the objective lens (S-TWIN). Images were recorded in STEM-HAADF and in annular bright-field (ABF) mode. The contrast in HAADF is proportional to the atomic number of the element. Therefore, in our catalysts Ru produces the brightest contrast. Relative content of Ti and Si in the mixed oxides was determined with the use of an energy dispersive micro-X-Ray fluorescence (XRF) spectrometer M4 TORNADO (Bruker) equipped with 2 anodes: a rhodium X-ray tube 50 kV/600 mA (30 W) and a tungsten X-Ray tube 50 kV/700 mA (35 W). For sample characterization, the X-rays Rhodium with a polycapillary lens enabling the excitation of an area of 200 μm was used. The detector was a silicon drift detector Si(Li) with <145 eV resolution at 100,000 cps (Mn Kα) working with a Peltier cooling (253°K). The measurement was done under vacuum (20 mbar). Quantitative analysis was performed using fundamental parameter (FP) (standardless). For each sample 36 points (of 200 μm) were analyzed.

### 3.5. Catalytic Tests

The catalytic performance of the supported catalysts was evaluated in the aqueous phase hydrogenation of LA to GVL. Prior to catalytic testing, the RuO_2_-based composite was placed in a U-tube quartz reactor and reduced at 400 °C for 2 h under H_2_ atmosphere (5% H_2_/Ar) with a heating rate of 2 °C min^−1^ and a flow rate of 10 mL min^−1^. Reaction was performed according to previously reported conditions for water by Michel et al. [13] with some modifications. In a typical experiment, a 50 mL stainless steel batch autoclave reactor, equipped with a magnetic drive stirring and a thermostatic bath for the temperature control, was loaded with 660 mg LA (5.68 mmol), 77.6 mg de-supported catalyst (substrate/metal molar ratio = 300), and 20 mL ultra-pure water. The mixture was purged three times with nitrogen at room temperature, then heated at 50 °C and charged with H_2_ to 50 bar. Reactions were run at a constant temperature of 50 °C for a certain reaction time (20 to 60 min) using a stirring speed of 750 rpm. At the end of the reaction, the reactor was cooled with cold water and the separation of the solid catalyst from the reaction mixture was performed through centrifugation and filtration. The composition of the reaction mixture was determined quantitatively by gas chromatography (GC). 50 µL of the separated liquid reaction products were diluted in 1 mL of chloroform in a GC vial. Benzaldehyde (1 wt.%) was used as an internal standard. Products were analyzed using a Shimadzu GC-17A gas chromatograph (GC) equipped with a UBFFAP302525 column (30 m × 0.25 mm × 0.25 µm) and a flame ionization detector (FID). The FID and injection port temperatures were 250 °C. LA conversion and GVL yield were determined based on the calibration curves (see Figure S13, ESI) using the following equations:

(2) $$\mathrm{Conversion}\;\left(\%\right)=\frac{\mathrm{moles}\;\mathrm{of}\;\mathrm{LA}\;\mathrm{reacted}}{\mathrm{total}\;\mathrm{moles}\;\mathrm{of}\;\mathrm{LA}}\times 100\;$$

(3) $$\mathrm{Yield}\;\left(\%\right)=\frac{\mathrm{moles}\;\mathrm{of}\;\mathrm{GVL}\;\mathrm{formed}}{\mathrm{total}\;\mathrm{moles}\;\mathrm{of}\;\mathrm{LA}}\times 100\;$$

Nuclear magnetic resonance (NMR) spectra were recorded at 298 K on a Bruker Avance III HD 300 NanoBay spectrometer equipped with a 5 mm broadband probe BBFO with Z-gradients, operating at 7.05 T field strength (300 MHz). ^1^H NMR chemical shifts are reported in ppm (δ) downfield from tetramethylsilane (TMS, in CDCl_3_). ^1^H NMR proved to be the most reliable method for the analysis of the intermediate product, 4-hydroxypentanoic acid (4-HPA), which is difficult to determine by GC. Typical profiles of the ^1^H NMR spectra of the isolated products and the reaction mixture are shown in Figures S9 and S10, ESI.

For the recyclability tests, the solid was recovered by centrifugation, washed with water (10 mL), and then with chloroform (2–10 mL). GC analysis of the washing organic phase was performed to check the absence of any organic compounds from the catalytic reaction. The solid was dried overnight under vacuum to remove chloroform before starting the next run.

## 4. Conclusions

In summary, a versatile template-directed colloidal approach for the fabrication of composition-tuned TiO_2_-SiO_2_ materials with controllable pore sizes and crystal phase compositions has been established by constructing a RaMeβCD-Pluronic F127-titanium dioxide-silica mesophase before template removal. The supramolecular template together with the silica network were found to affect not only the structural and textural characteristics of the support but also the morphology of RuO_2_ nanostructures which underwent shape transformation from spherical to plate-like, lozenge-like, and rod-like particles. When utilized as catalysts, Ru particles supported on mesoporous TiO_2_-SiO_2_ mixed oxides globally displayed higher catalytic activity in the hydrogenation of levulinic acid (LA) to ɣ-valerolactone (GVL) compared to Ru deposited on mesoporous TiO_2_. The enhanced catalytic performance was attributed to the larger surface area and higher porosity of TiO_2_-SiO_2_ support, which promoted uniform dispersion of Ru nanoparticles, reducing particle agglomeration and mass transfer limitations during the catalytic process. Moreover, the activity was improved by using elongated Ru structures as opposed to spherical Ru particles. Thus, a 100% conversion of LA together with a 99% yield in GVL was achieved at 50 °C under 50 bar H_2_ in one hour on a Ru@TiO_2_-SiO_2_ 3:3-ns catalyst in the presence of water. Importantly, this catalyst demonstrated high stability in this reaction and could be reused at least five times without an obvious loss of activity. Its robustness was ascribed to the confinement effects of nanocrystals in a highly porous TiO_2_-SiO_2_ matrix, as well as the strong metal support interactions. Altogether, this work successfully proves the important role of RaMeβCD-based supramolecular assemblies in the fabrication of composition-tuned heterogeneous catalysts with enhanced catalytic performance in aqueous phase hydrogenation reactions.

## Figures and Tables

**Table 1:** Structural and textural characteristics of the TiO_2_ materials and TiO_2_-SiO_2_ composites after calcination at 500 °C.

Sample	S_BET_^a^ (m^2^ g^−1^)	V_cum_^b^ (cm^3^ g^−1^)	PS ^c^ (nm)	Crystallite Size ^d^ (nm)		
				Anatase TiO_2_	Brookite TiO_2_	Rutile TiO_2_
TiO_2_-sg	21 ± 1	0.030 ± 0.002	<2.0 ± 0.4	36.4 ± 2.9	18.7 ± 1.5	60.4 ± 4.8
TiO_2_-ns	80 ± 4	0.20 ± 0.01	7.4 ± 1.5	11.0 ± 0.9	4.5 ± 0.4	11.5 ± 0.9
TiO_2_-SiO_2_ 3:1-ns	169 ± 8	0.49 ± 0.02	11.1 ± 2.2	10.3 ± 0.8	4.4 ± 0.4	9.5 ± 0.8
TiO_2_-SiO_2_ 3:2-ns	198 ± 10	0.93 ± 0.05	18.1 ± 3.6	6.2 ± 0.5	2.7 ± 0.2	-
TiO_2_-SiO_2_ 3:3-ns	193 ± 10	1.17 ± 0.06	24.0 ± 4.8	6.1 ± 0.5	2.7 ± 0.2	-

^a^ Specific surface area determined in the relative pressure range 0.1–0.25; ^b^ cumulative pore volume and ^c^ average pore size resulting from BJH calculations, ^d^ crystallite size calculated using the Scherrer equation.

**Table 2:** Characteristics of supported RuO_2_@TiO_2_ and RuO_2_@TiO_2_-SiO_2_ catalysts after thermal treatment at 400 °C.

Sample	S_BET_^a^ (m^2^ g^−1^)	V_cum_^b^ (cm^3^ g^−1^)	PS ^c^ (nm)	RuO_2_ Size ^d^ (nm)
RuO_2_@TiO_2_-sg	8 ± 0.4	0.015 ± 0.00	<2.0 ± 0.4	15.8 ± 1.6
RuO_2_@TiO_2_-ns	65 ± 3.2	0.18 ± 0.01	6.1 ± 1.2	9.9 ± 1.0
RuO_2_@TiO_2_-SiO_2_ 3:1-ns	162 ± 8.1	0.43 ± 0.021	10.6 ± 2.1	9.0 ± 0.9
RuO_2_@TiO_2_-SiO_2_ 3:2-ns	138 ± 6.9	0.50 ± 0.03	15.0 ± 3.0	-
RuO_2_@TiO_2_-SiO_2_ 3:3-ns	156 ± 7.8	0.72 ± 0.04	15.1 ± 3.0	-

^a^ specific surface area determined in the relative pressure range 0.1–0.25, ^b^ cumulative pore volume and ^c^ average pore size resulting from Barrett, Joyner, and Halenda (BJH) calculations; ^d^ RuO_2_ crystallite size determined using the Scherrer equation.

**Table 3:** Catalytic hydrogenation of LA to GVL with the different Ru-based catalysts ^a^.

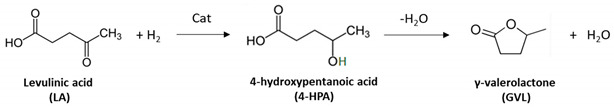						
Entry	Catalyst	TiO_2_ (%) ^b^	SiO_2_ (%) ^b^	LA Conversion (%) ^c^	GVL Yield (%) ^c^	Productivity (mol_GVL_ g^−1^_metal_ h^−1^) ^d^
1	TiO_2_-ns	100	0	<3	0	-
2	TiO_2_-SiO_2_ 3:3-ns	66	34	<3	0	-
3	Ru@TiO_2_-sg	100	0	65	46	4.0434
4	Ru@TiO_2_-ns	100	0	79	58	5.0983
5	Ru@TiO_2_-SiO_2_ 3:1-ns	87	13	82	75	6.5926
6	Ru@TiO_2_-SiO_2_ 3:2-ns	76	24	88	76	6.6805
7	Ru@TiO_2_-SiO_2_ 3:3-ns	66	34	91	78	6.8563
8	Ru@TiO_2_-SiO_2_ 3:3-sg	68	32	73	56	4.9225
9	Ru@C	-	-	99	66	5.8015

^a^ Reaction conditions: 0.66 g LA (5.68 mmol), 77.6 mg supported catalyst (0.019 mmol Ru), 50 bar H_2_, 20 mL H_2_O, temperature 323 K, and reaction time 20 min. ^b^ Mass % of oxide determined by XRF. ^c^ Determined by GC using Equations (2) and (3) (see experimental part) and benzaldehyde as an internal standard. ^d^ Productivity: moles of GVL produced per g of Ru per hour.

## Data Availability

The data presented in this study are available in article and supplementary material.

## Supplementary Materials

The following are available online at https://www.mdpi.com/1422-0067/22/4/1721/s1, Figure S1: DLS plots of micellar solutions prepared with 3% *w*/*v* F127 (A), 5% *w*/*v* F127 (B) and 15% *w*/*v* F127 (C) and increasing concentrations of RaMeβCD from 10 mg mL^−1^ to 110 mg mL^−1^, Figure S2: N2-adsorption isotherms and corresponding pore size distributions (inset) of SiO_2_-sg and SiO_2_-ns, Figure S3: XRD patterns of TiO_2_-SiO_2_ 3:3-sg (a) and RuO_2_@TiO_2_-SiO_2_ 3:3-sg (b), Figure S4: HAADF-STEM image (a) and corresponding EDX elemental mapping of Ti (b), Si (c), Ru (d) and O (e) of RuO_2_@TiO_2_-SiO_2_ 3:1-ns catalyst, Figure S5: Representative HR-TEM images of RuO_2_@TiO_2_-SiO_2_ 3:1-ns catalyst, Figure S6: Representative HAADF-STEM images of RuO_2_@TiO_2_-SiO_2_ 3:2-ns catalyst showing formation of rod-shaped crystals of 50−100 nm length and 20−40 nm width together with corresponding EDX elemental mapping of Ti (e), Si (f), Ru (g) and O (h), Figure S7: HAABF- (a) and HAADF-STEM (b) images and corresponding EDX elemental mapping of Ti (c), Si (d), Ru (e) and O (f) (B) of RuO2@TiO2-SiO23:3-ns catalyst, Figure S8: TPR patterns of RuO_2_@TiO_2_-SiO_2_ 3:3-sg (a) and RuO2@TiO2-SiO2 3:3-ns (b), Figure S9: ^1^H NMR (300MHz, CDCl3) spectra of (A) LA and (B) GVL, Figure S10: ^1^H NMR (300MHz, CDCl3) spectrum of reaction mixture in the aqueous phase hydrogenation of LA to GVL showing formation of 4-hydroxypentanoic acid (4-HPA) as intermediate, Figure S11: Comparison of LA conversions and GVL yields obtained with Ru catalysts supported on different mixed oxides prepared without template, Figure S12: Observed (red scatter) and calculated (black line) XRD patterns resulting from pattern matching of TiO_2_-sg (A) and TiO_2_-SiO_2_ 3:3-ns (B), Figure S13. Calibration curves of LA (A) and GVL (B) determined by GC using benzaldehyde as internal standard, Table S1: Textural characteristics of SiO_2_-sg and SiO_2_-ns supports after calcination at 500 °C, Table S2: Textural characteristics of sol-gel and nanostructured Al_2_O_3_ and Al_2_O_3_-based mixed oxides after calcination at 500 °C.

## Abbreviations

CD	Cyclodextrin
RaMeβCD	Randomly methylated β-cyclodextrin
GVL	γ-valerolactone
4-HPA	4-hydroxypentanoic acid
LA	Levulinic acid
TEOS	Tetraethyl orthosilicate
Ti(O^i^Pr)_4_	Titanium isopropoxyde
DLS	Dynamic light scattering
JCPDS	Joint committee on powder diffraction standards
XRD	X-ray diffraction
S_BET_	Surface area determined by the Brunauer, Emmett, and Teller method
BJH	Barrett, Joyner, and Halenda
TPR	Temperature programmed reduction
XRF	X-ray fluorescence
HR-TEM	High-resolution transmission electron microscopy
HAABF-STEM	High-angle annular bright-field scanning TEM
HAADF-STEM	High-angle annular dark-field scanning TEM
EDS	Energy dispersive X-ray spectrometry
ICP-OES	Inductive coupled plasma optical emission spectroscopy
NMR	Nuclear magnetic resonance
GC	Gas chromatography
