# Supplementary material for: Robust Ruthenium Catalysts Supported on Mesoporous Cyclodextrin-Templated TiO2-SiO2 Mixed Oxides for the Hydrogenation of Levulinic Acid to γ-Valerolactone
Source: Int J Mol Sci. 2021 Feb 9;22(4):1721. doi: 10.3390/ijms22041721 (PMC7915766; doi:10.3390/ijms22041721)
Supplement: Supplementary file 1 [file ijms-22-01721-s001.pdf]

Electronic Supplementary Information for

Robust Ruthenium Catalysts Supported on Mesoporous Cyclodextrin-Templated TiO_2-SiO_2 Mixed Oxides for the Hydrogenation of Levulinic Acid to γ-Valerolactone

Cédric Decarpigny [1], Sébastien Noël [1], Ahmed Addad [2], Anne Ponchel [1], Eric Monflier [1] and Rudina Bleta [1,*]

[1] University Artois, CNRS, Centrale Lille, ENSCL, Univ. Lille, UMR 8181-UCCS-Unité de Catalyse et Chimie du Solide, F-62300 Lens, France; cedric.decarpigny@univ-artois.fr (C.D.); sebastien.noel@univ-artois.fr (S.N.); anne.ponchel@univ-artois.fr (A.P.); eric.monflier@univ-artois.fr (E.M.); rudina.bleta@univ-artois.fr (R.B.)

[2] University Lille, CNRS, INRA, ENSCL, UMR 8207-UMET-Unité Matériaux et Transformations, F-59000 Lille, France; ahmed.addad@univ-lille.fr (A.A.)

* Correspondence: rudina.bleta@univ-artois.fr; Tel.: (+33) 3 21 79 17 40

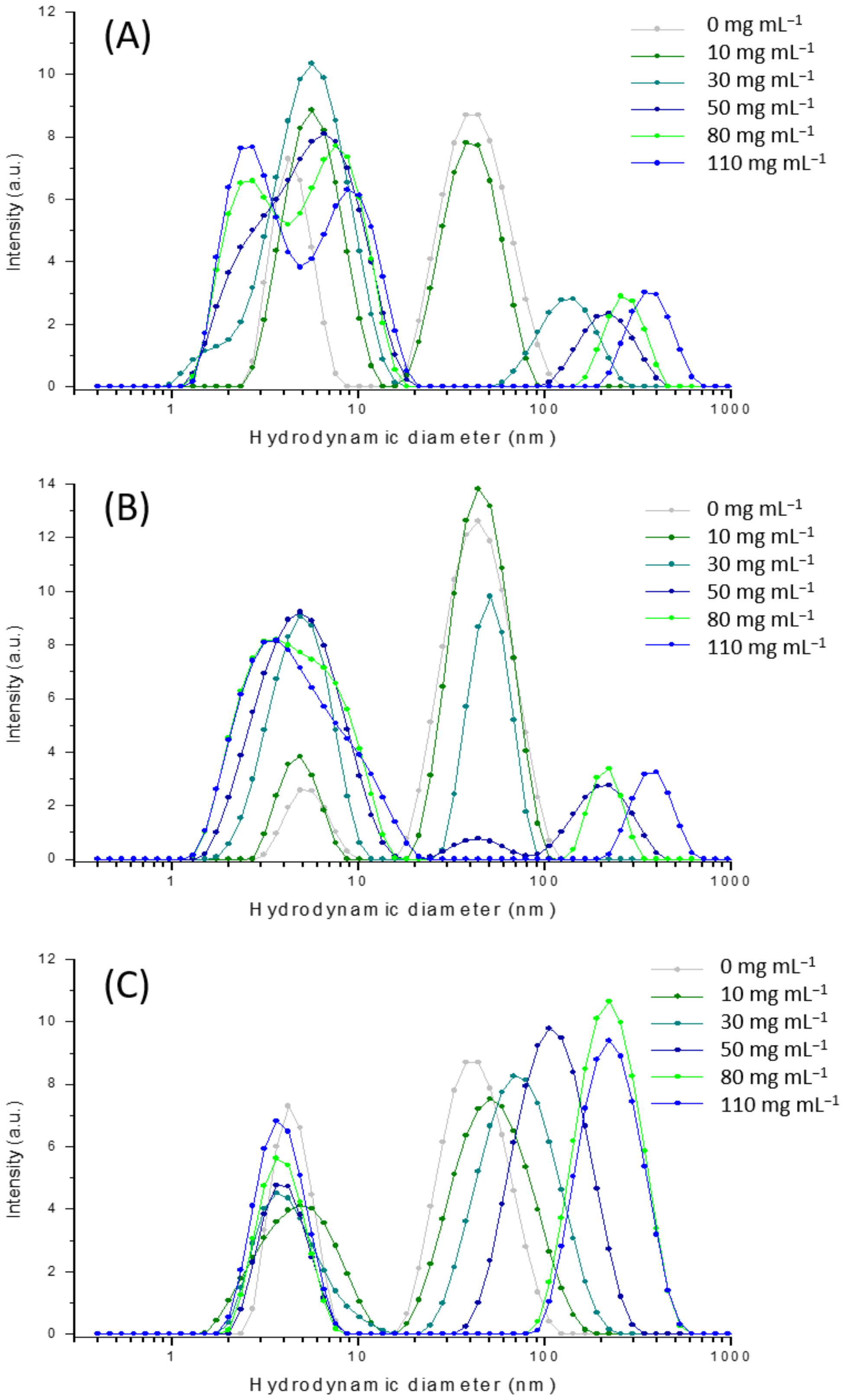

Figure S1. DLS plots of micellar solutions prepared with 3 % w/v F127 (A), 5 % w/v F127 (B) and 15 % w/v F127 (C) and increasing concentrations of RaMeβCD from 10 mg ml^{-1} to 110 mg ml^{-1}.

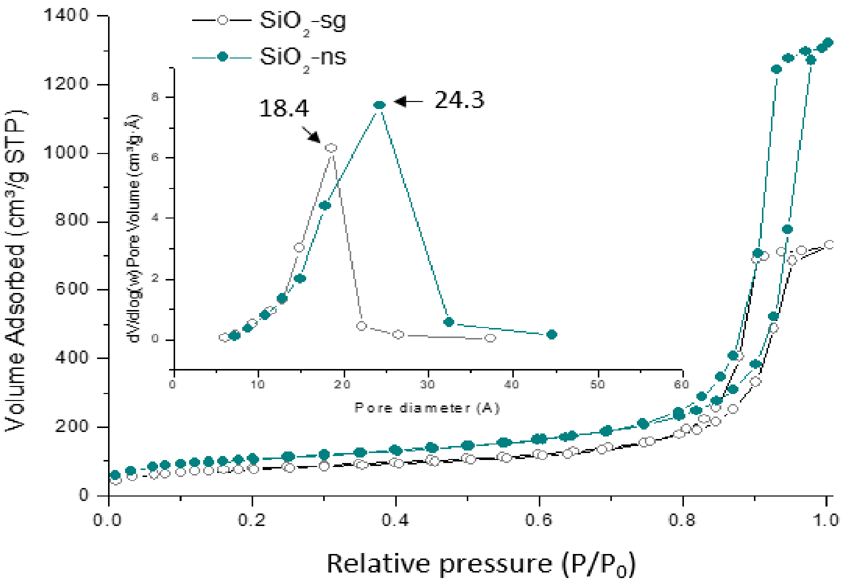

Figure S2. N_2-adsorption isotherms and corresponding pore size distributions (inset) of SiO_2-sg and SiO_2-ns.

Table S1. Textural characteristics of SiO_2-sg and SiO_2-ns supports after calcination at 500 °C.

Sample	S_{BET}[a] (m^2 g^{-1})	V_{cum}[b] (cm^3 g^{-1})	PS[c] (nm)
SiO_2-sg	281±14	1.12±0.06	18.4±3.7
SiO_2-ns	381±19	2.04±0.10	24.3±4.9

[a]specific surface area determined in the relative pressure range 0.1-0.25, [b]cumulative pore volume and [c]average pore size resulting from BJH calculations.

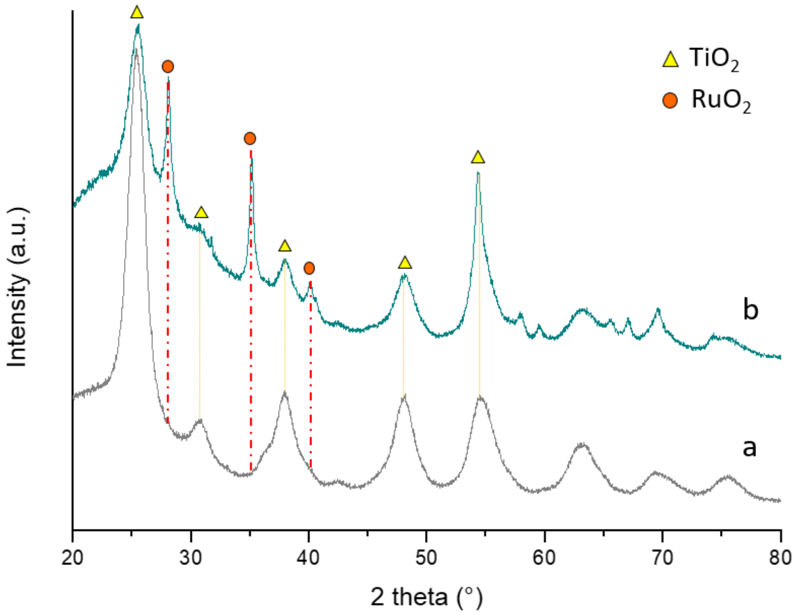

Figure S3. XRD patterns of TiO_2-SiO_2 3:3-sg (a) and RuO_2@TiO_2-SiO_2 3:3-sg (b).

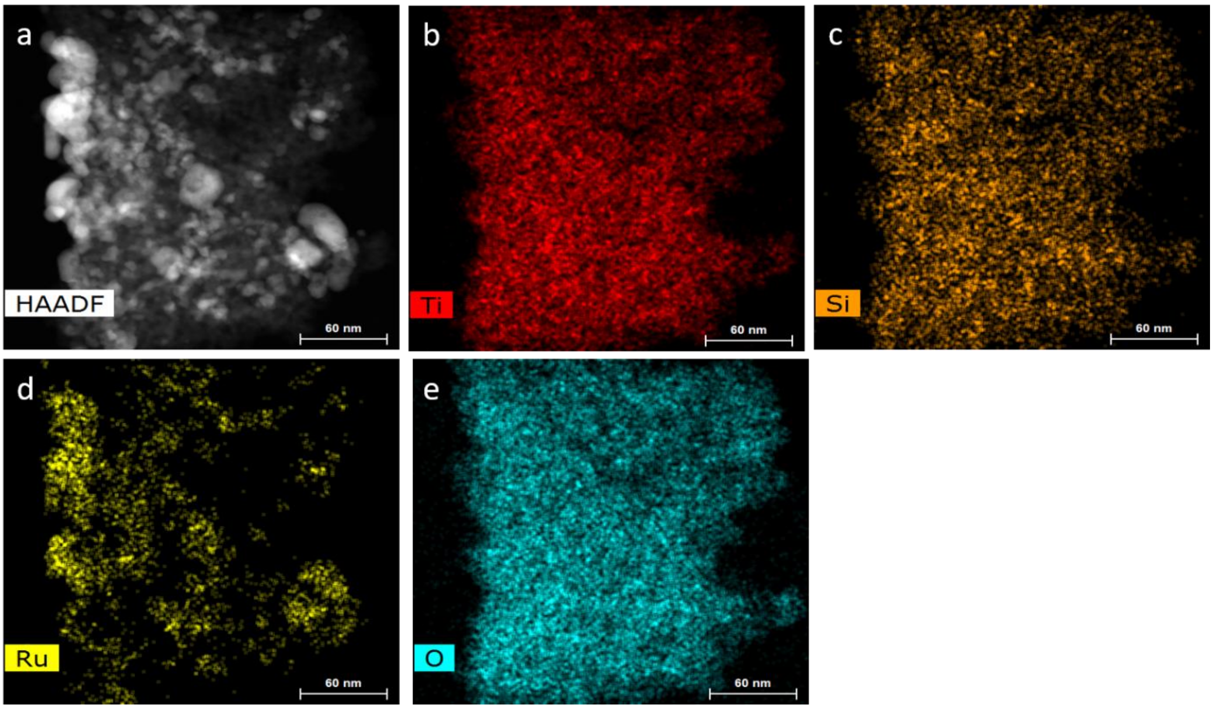

Figure S4. HAADF-STEM image (a) and corresponding EDX elemental mapping of Ti (b), Si (c), Ru (d) and O (e) of RuO_2@TiO_2-$SiO_2$3:1-ns catalyst.

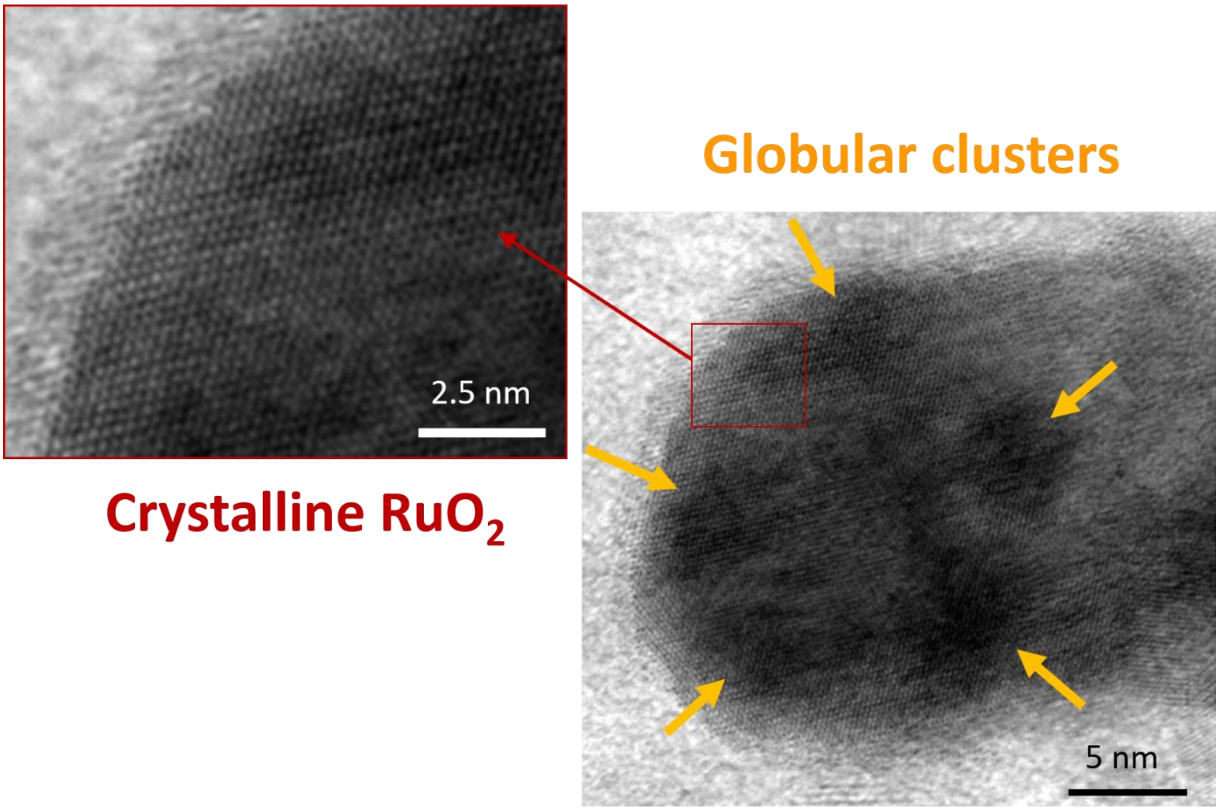

Figure S5. Representative HR-TEM images of RuO_2@TiO_2-$SiO_2$3:1-ns catalyst.

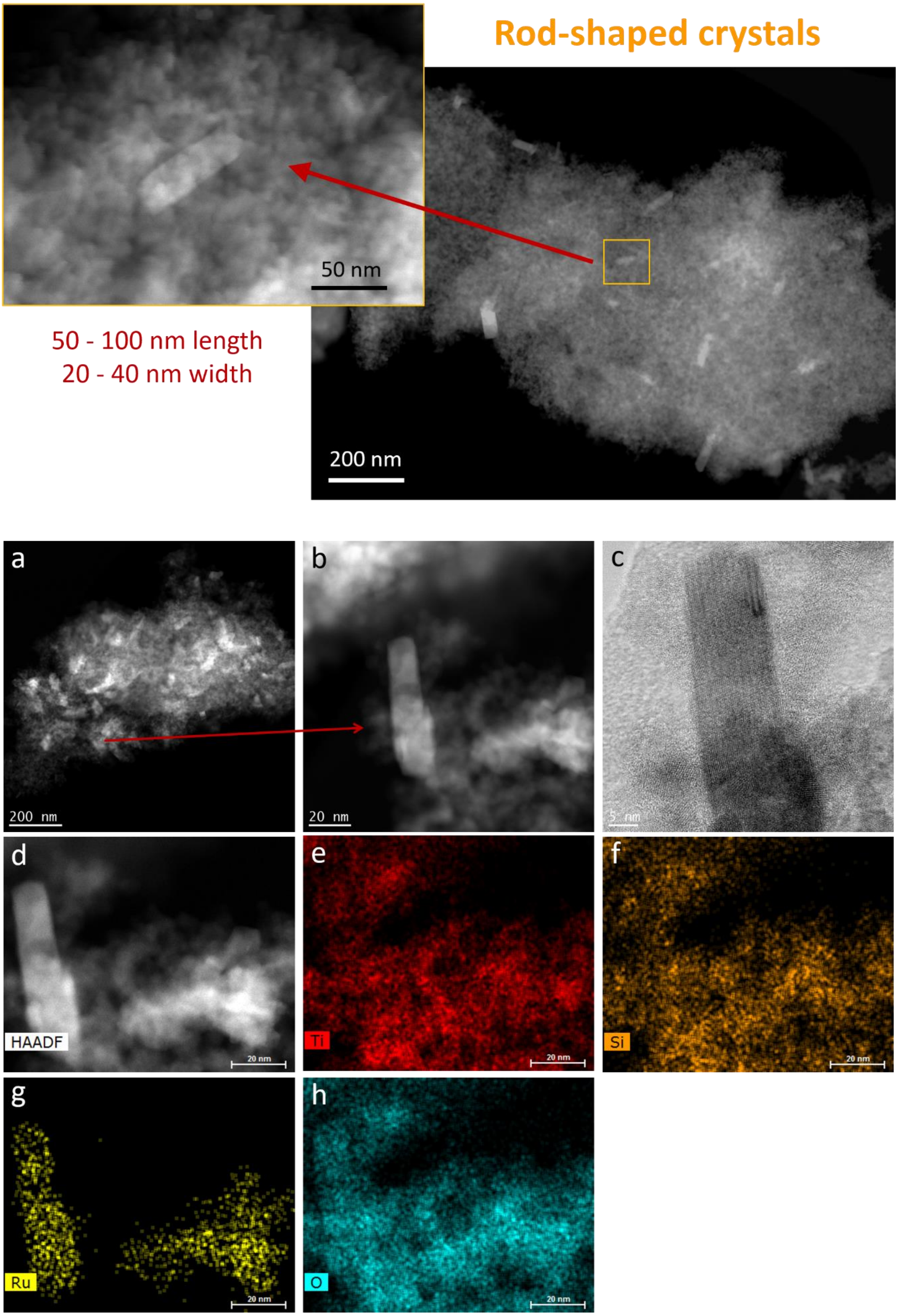

Figure S6. Representative HAADF-STEM images of RuO_2@TiO_2-$SiO_2$3:2-ns catalyst showing formation of rod-shaped crystals of 50-100 nm length and 20-40 nm width together with corresponding EDX elemental mapping of Ti (e), Si (f), Ru (g) and O (h).

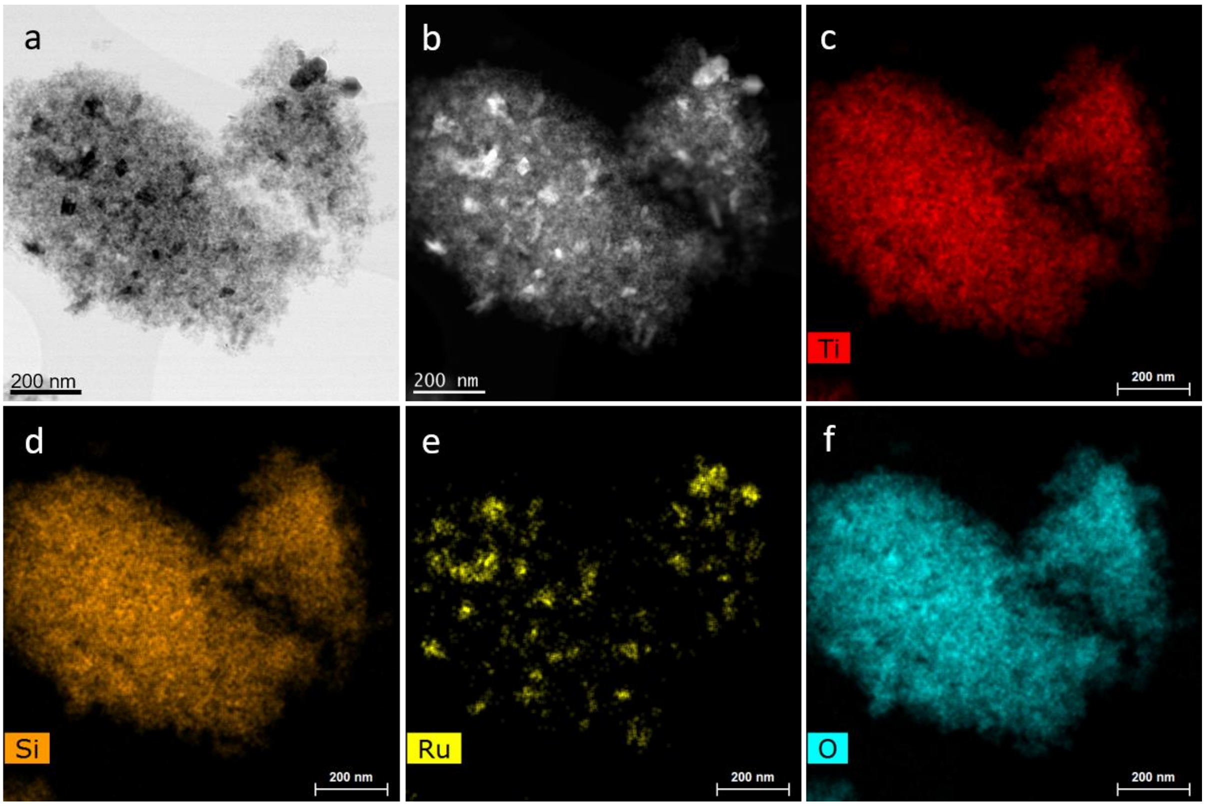

Figure S7. HAABF- (a) and HAADF-STEM (b) images and corresponding EDX elemental mapping of Ti (c), Si (d), Ru (e) and O (f) (B) of $RuO_2@TiO_2$-$SiO_2$3:3-ns catalyst.

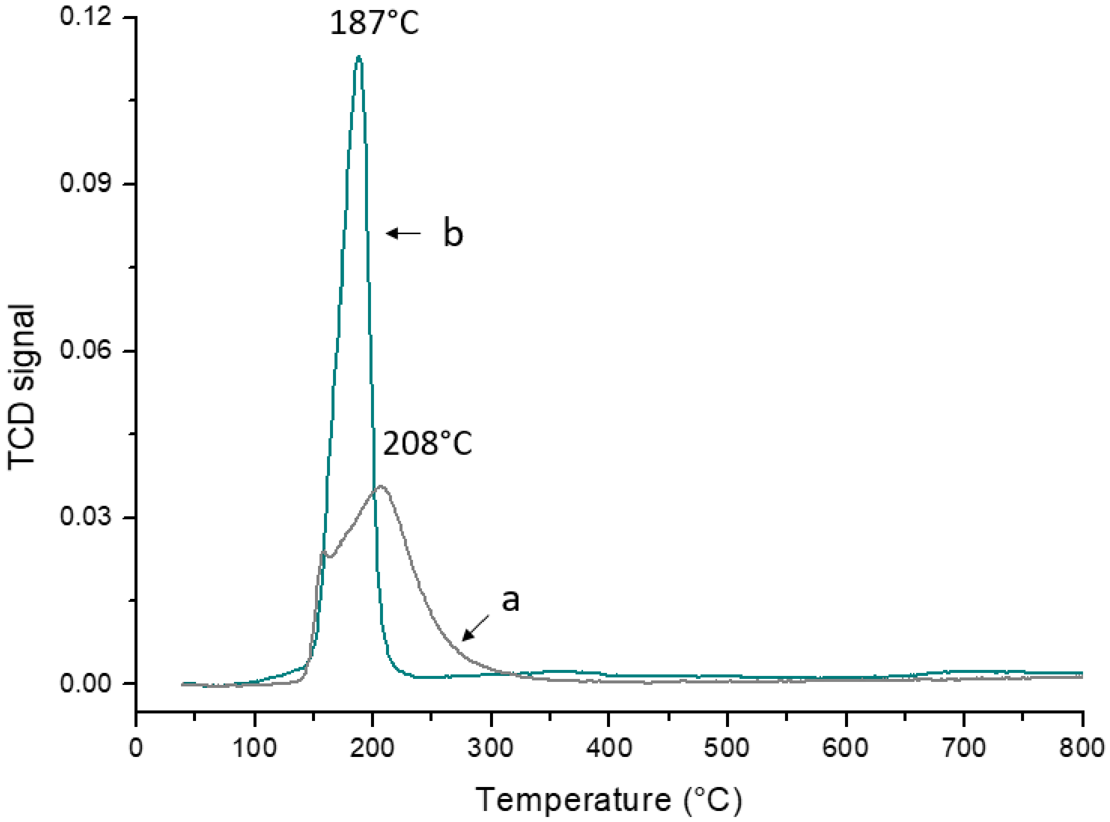

Figure S8. TPR patterns of $RuO_2@TiO_2$-SiO_2 3:3-sg (a) and $RuO_2@TiO_2$-SiO_2 3:3-ns (b).

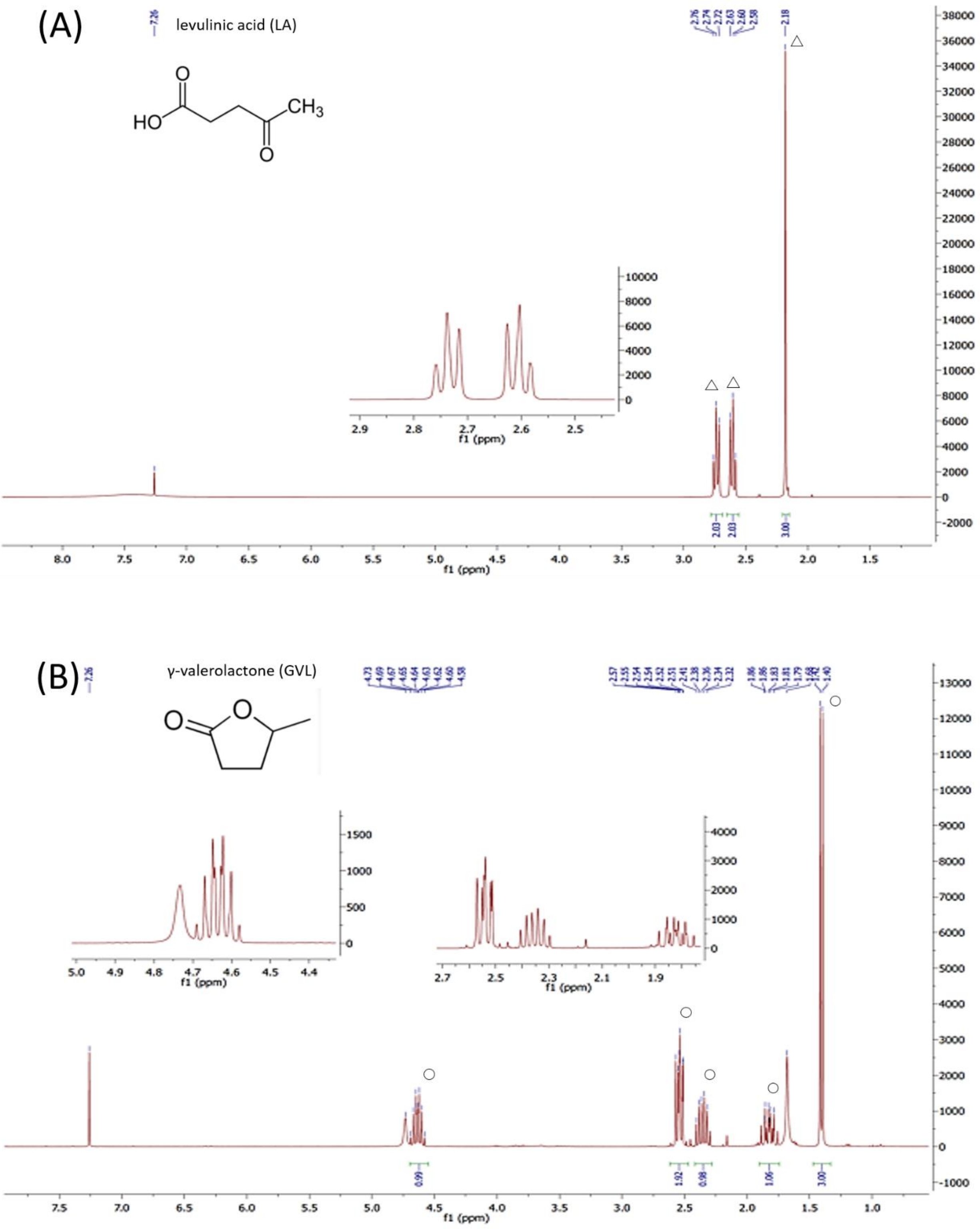

Figure S9. ¹H NMR (300MHz, $CDCl_3$) spectra of (A) LA and (B) GVL.

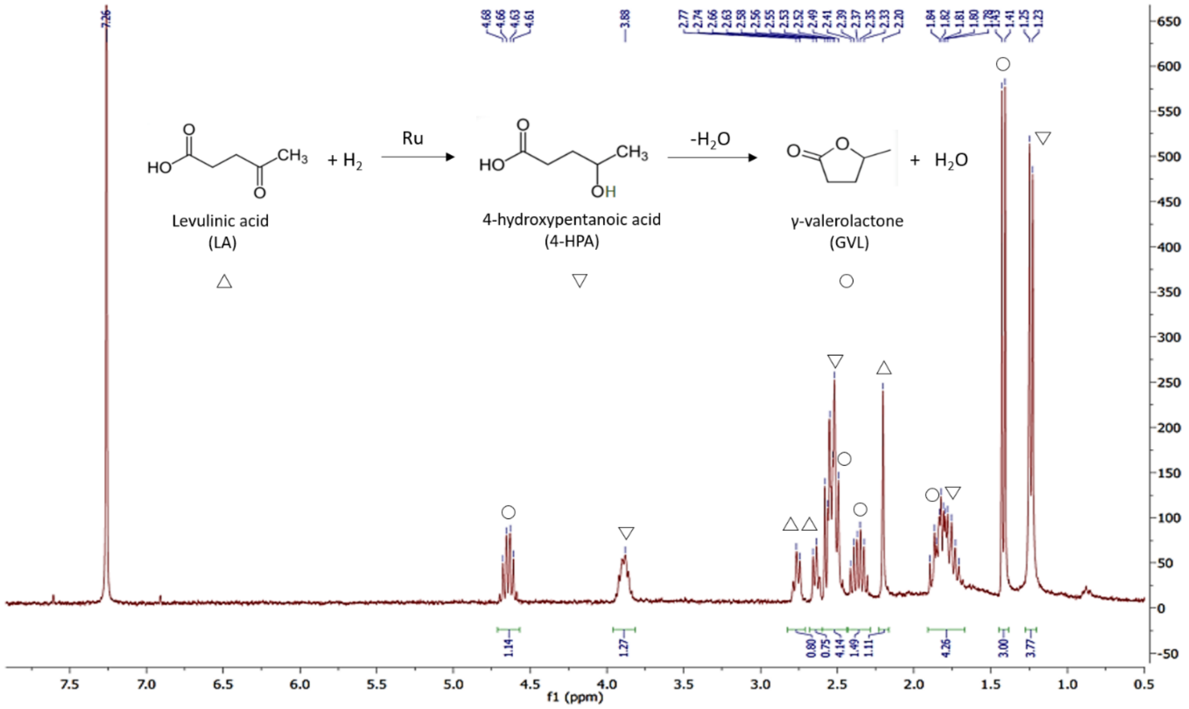

Figure S10. ^1H NMR (300MHz, $CDCl_3$) spectrum of reaction mixture in the aqueous phase hydrogenation of LA to GVL showing formation of 4-hydroxypentanoic acid (4-HPA) as intermediate.

Table S2. Textural characteristics of sol-gel and nanostructured Al_2O_3 and Al_2O_3-based mixed oxides after calcination at 500 °C.

Sample	S_{BET}[a] ($m^2\ g^{-1}$)	V_{cum}[b] ($cm^3\ g^{-1}$)	PS[c] (nm)
Al_2O_3-sg	97±5	0.34±0.02	11.2±2.2
Al_2O_3-ns	236±12	0.67±0.03	17.3±3.5
TiO_2-Al_2O_3 3:3-sg	192±10	0.35±0.02	6.2±1.2
TiO_2-Al_2O_3 3:3-ns	183±9	0.69±0.03	10.7±2.1
SiO_2-Al_2O_3 3:3-sg	436±22	1.05±0.05	9.5±1.9
SiO_2-Al_2O_3 3:3-ns	373±19	1.41±0.07	15.0±3.0

[a]specific surface area determined in the relative pressure range 0.1-0.25, [b]cumulative pore volume and [c]average pore size resulting from BJH calculations.

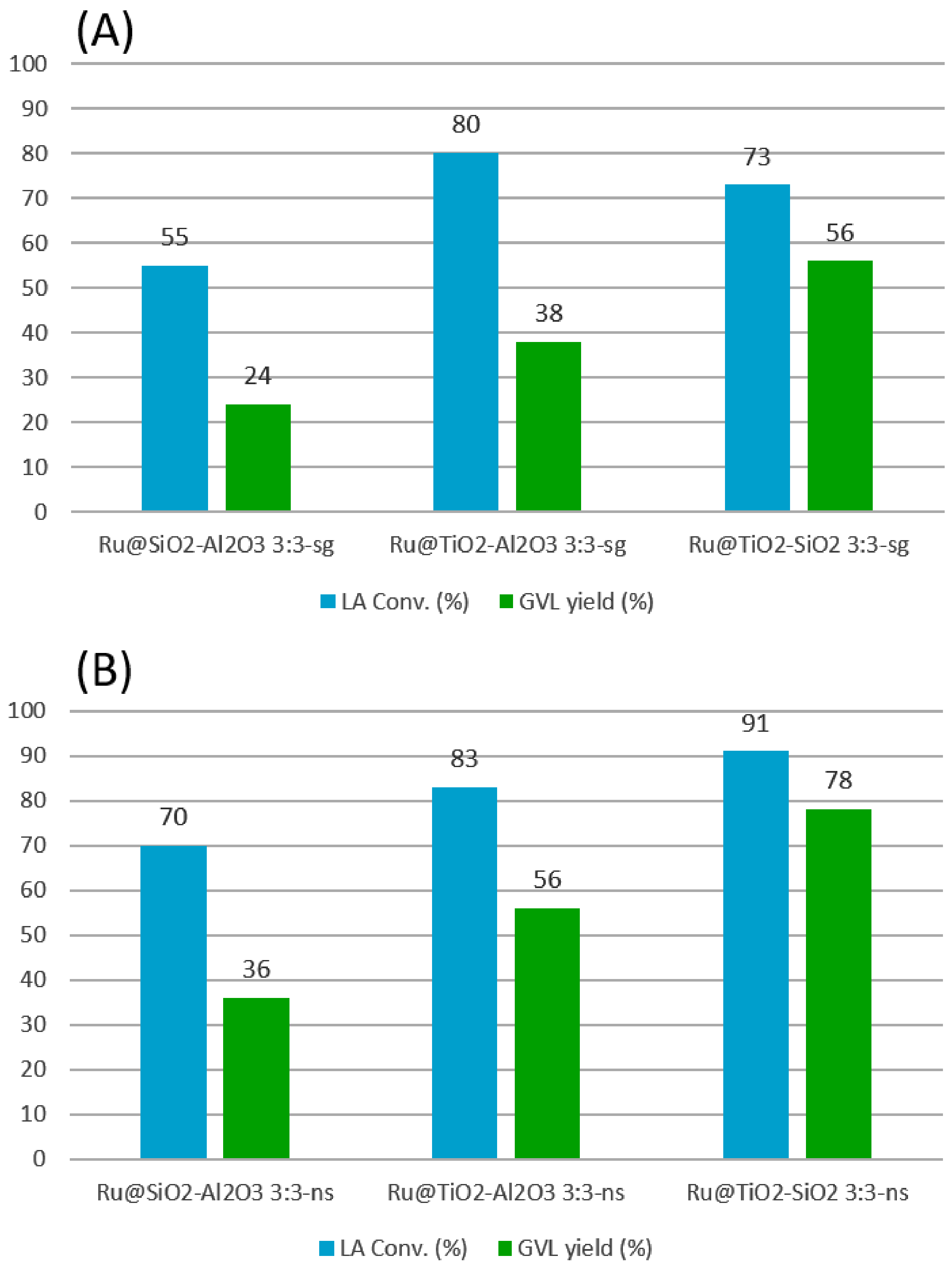

Figure S11. Comparison of LA conversions and GVL yields obtained with Ru catalysts supported on different mixed oxides prepared without template (A) and with the RaMeβCD/F127 supramolecular assemblies (B). Reaction conditions: 0.66 g LA (5.68 mmol), 77.6 mg supported catalyst (0.019 mmol Ru), 50 bar H_2, 20 ml H_2O, temperature 323 K, reaction time 20 min.

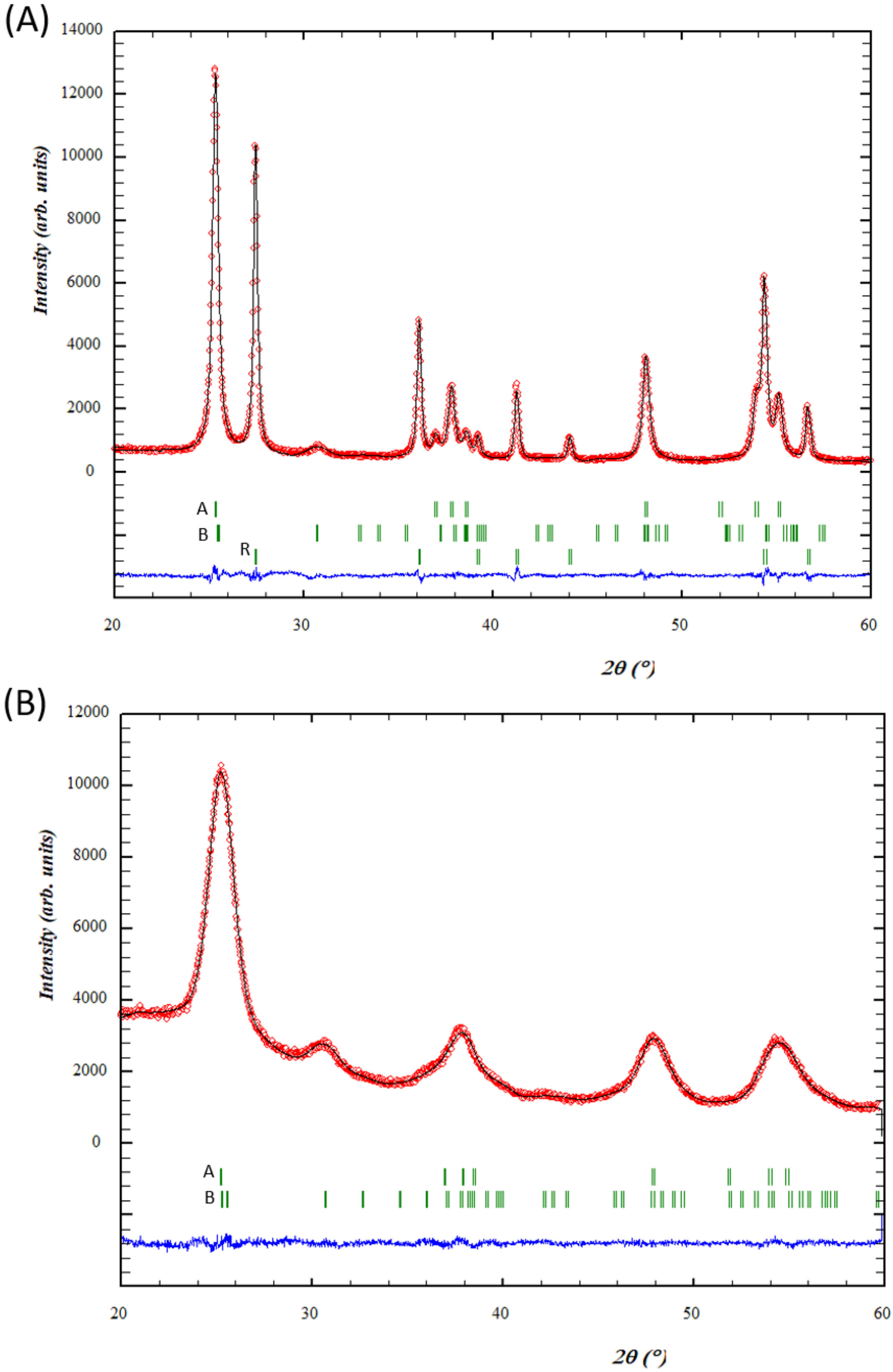

Figure S12. Observed (red scatter) and calculated (black line) XRD patterns resulting from pattern matching of TiO_2-sg (A) and TiO_2-SiO_2 3:3-ns (B). Green lines: JCPDS of anatase (A), brookite (B) and rutile (R). Blue line: difference profile between the experimental and the calculated patterns. The χ^2 obtained after refinement were 2.54 for TiO_2-sg and 1.48 for TiO_2-SiO_2 3:3-ns.

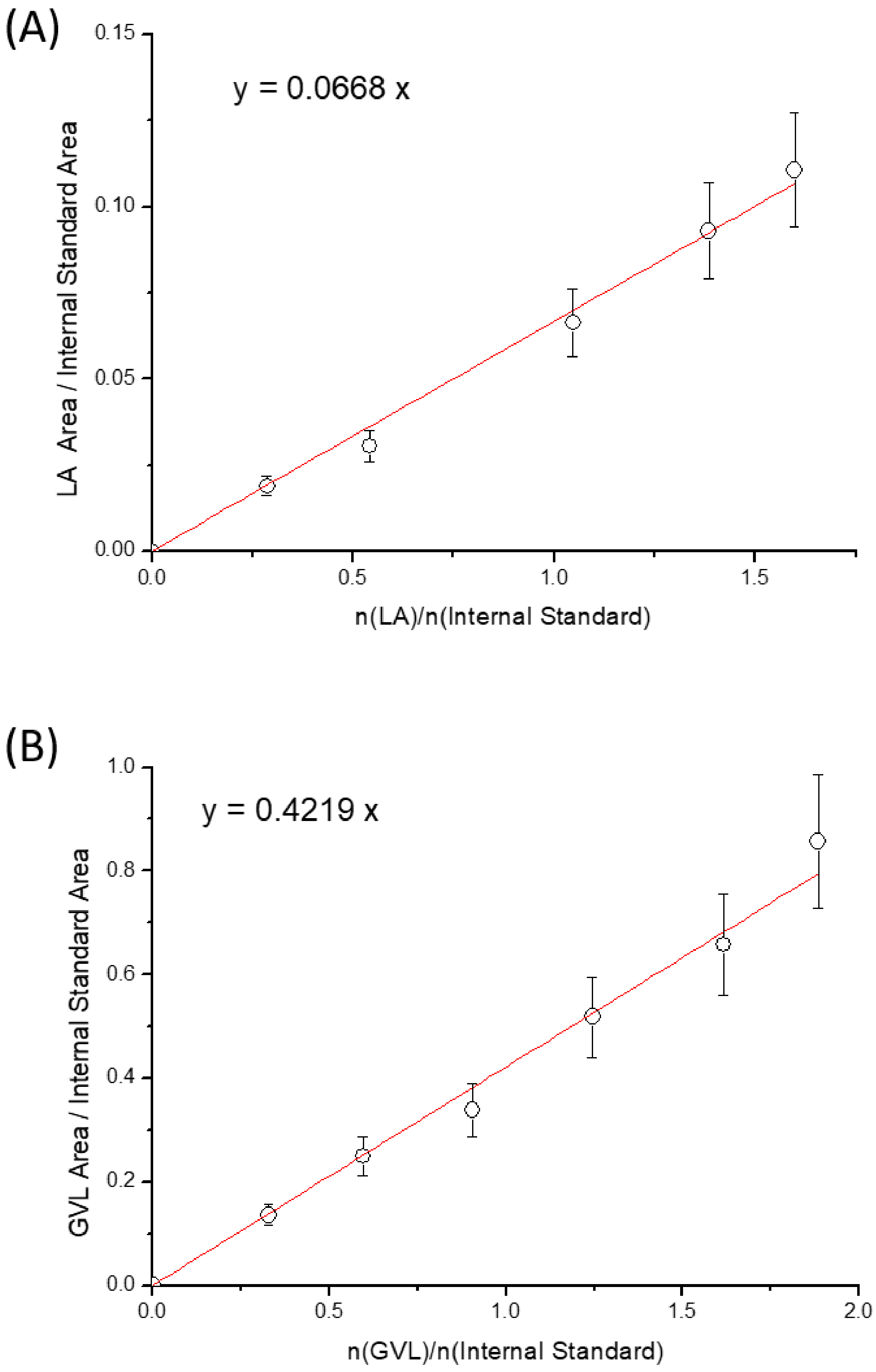

Figure S13. Calibration curves of LA (A) and GVL (B) determined by GC using benzaldehyde as internal standard.
